# Does Integrated Management of Childhood Illness (IMCI) Training Improve the Skills of Health Workers? A Systematic Review and Meta-Analysis

**DOI:** 10.1371/journal.pone.0066030

**Published:** 2013-06-12

**Authors:** Duyen Thi Kim Nguyen, Karen K. Leung, Lynn McIntyre, William A. Ghali, Reg Sauve

**Affiliations:** 1 Department of Community Health Sciences, Faculty of Medicine, University of Calgary, Alberta, Canada; 2 Population Health and Inequities Research Centre, University of Calgary, Alberta, Canada; 3 Population Health Intervention Research Network, University of Calgary, Alberta, Canada; 4 Department of Family Medicine, Faculty of Medicine, University of Alberta, Alberta, Canada; 5 Department of Medicine, Faculty of Medicine, University of Calgary, Alberta, Canada; 6 Institute for Public Health, University of Calgary, Alberta, Canada; 7 Department of Paediatrics, Faculty of Medicine, University of Calgary, Alberta, Canada; Tehran University of Medical Sciences, Iran (Islamic Republic of)

## Abstract

**Background:**

An estimated 6.9 million children die annually in low and middle-income countries because of treatable illneses including pneumonia, diarrhea, and malaria. To reduce morbidity and mortality, the Integrated Management of Childhood Illness strategy was developed, which included a component to strengthen the skills of health workers in identifying and managing these conditions. A systematic review and meta-analysis were conducted to determine whether IMCI training actually improves performance.

**Methods:**

Database searches of CIHAHL, CENTRAL, EMBASE, Global Health, Medline, Ovid Healthstar, and PubMed were performed from 1990 to February 2013, and supplemented with grey literature searches and reviews of bibliographies. Studies were included if they compared the performance of IMCI and non-IMCI health workers in illness classification, prescription of medications, vaccinations, and counseling on nutrition and admistration of oral therapies. Dersminion-Laird random effect models were used to summarize the effect estimates.

**Results:**

The systematic review and meta-analysis included 46 and 26 studies, respectively. Four cluster-randomized controlled trials, seven pre-post studies, and 15 cross-sectional studies were included. Findings were heterogeneous across performance domains with evidence of effect modification by health worker performance at baseline. Overall, IMCI-trained workers were more likely to correctly classify illnesses (RR = 1.93, 95% CI: 1.66–2.24). Studies of workers with lower baseline performance showed greater improvements in prescribing medications (RR = 3.08, 95% CI: 2.04–4.66), vaccinating children (RR = 3.45, 95% CI: 1.49–8.01), and counseling families on adequate nutrition (RR = 10.12, 95% CI: 6.03–16.99) and administering oral therapies (RR = 3.76, 95% CI: 2.30–6.13). Trends toward greater training benefits were observed in studies that were conducted in lower resource settings and reported greater supervision.

**Conclusion:**

Findings suggest that IMCI training improves health worker performance. However, these estimates need to be interpreted cautiously given the observational nature of the studies and presence of heterogeneity.

## Introduction

Although the worldwide child mortality rate has declined by two-fifths since 1990 [1], an estimated 6.9 million children under the age of five still die annually in low and middle income countries (LMIC) because of preventable and treatable illnesses including pneumonia, diarrheal disease, malaria, and underlying malnutrition [2], [3]. In an effort to reduce pediatric morbidity and mortality, the World Health Organization (WHO) and other technical partners developed the Integrated Management of Childhood Illness (IMCI), an evidence-based strategy comprised of strengthening the skills of health workers, the health system, and family and community health practices [4], [5]. More than 100 countries have adopted components of IMCI, and in particular, the health worker case management guidelines for assessment and treatment of sick children, preventive care, and counseling of caregivers [6], [7]. Recognizing that many health workers may have limited pre-service training [8], and that sick children often present with undifferentiated and overlapping symptoms [9], IMCI health worker training provides a short-course, syndrome-based approach for identifying and managing illnesses. Accelerating the development of health worker competencies is essential, as both the density of health workers and the quality of care are independent predictors of child survival [10], [11].

IMCI case management consists of an integrated set of interventions with established survival benefits including the provision of vaccinations, antimicrobials for infectious diseases, and counseling on malnutrition and oral rehydration therapy [12]. Despite nearly two decades since its inception, understanding the effects of IMCI on the diagnosis and equitable management of illnesses such as pneumonia and diarrhea remain key research priorities [13]. Early analyses including a systematic review by Amaral and Victora [14], which provided a narrative summary of algorithms and worker performance until 2006, concluded that training improved assessment, communication, and rational antibiotic use [15]. However, recent evaluations suggest that IMCI has fallen somewhat short of expectations given the low population coverage, fragmented health systems, and weak community health promotion [16], [17], [18], [19]. Furthermore, it remains unclear whether training consistently improved skills in other domains such as vaccinations and nutrition counseling, and if so, the magnitude of these benefits. Underpinning these issues, however, is the adequacy of IMCI implementation. Factors such as the presence of sufficient equipment, essential drugs, supervisory visits, and duration of IMCI training not only determine health worker performance but also the level of intervention coverage that is ultimately achievable [20], [21]. To date, only one systematic review by Rowe and colleagues [22] has explored implementation adequacy as a confounding factor on health worker performance, albeit within a slightly different context of shortening IMCI training.

Therefore, we conducted a systematic review and meta-analysis of whether IMCI training improves health worker performance in five domains selected based on known survival benefits: classifying illnesses, prescribing appropriate medications, providing vaccinations, counseling caregivers on adequate nutrition, and instructing caregivers on administering oral therapies [12]. We extended the review by Amaral and Victora [14] by providing an updated literature synthesis and quantitative evaluation of performance, and we complemented the work by Rowe and colleagues [22] by examining the confounding effects of implementation adequacy, study design and methodological quality. We further assessed the contributions of these factors to heterogeneity, which is an expected element in meta-analyses of public health interventions [23].

## Methods

### Search Strategy

A systematic review and meta-analysis were conducted using a predetermined protocol (Text S1), and in accordance with the Preferred Reporting Items for Systematic Reviews and Meta-analyses (PRISMA) guidelines (Text S2). Database searches of MEDLINE, EMBASE, Ovid HealthStar, Global Health, CENTRAL, CINAHL and PubMed were performed without language restrictions. Due to the development of IMCI in the mid-1990s [4], [5], our search included studies published from 1990 to February 2013. To identify pertinent, unpublished grey literature, we conducted supplemental searches using the websites of the WHO Library Database (WHOLIS), WHO Department of Child and Adolescent Health and Development and its regional offices, IMCI Multi-Country Evaluation (MCE) research group, Department for International Development (DFID), United States Agency for International Development (USAID), Proquest, Thesis Canada Portal, and Scopus. The 2005 Health Policy and Planning journal supplement on IMCI as well as the bibliographies of literature reviews and key articles were reviewed to locate additional publications. Research teams were further contacted regarding their knowledge of any missed or ongoing studies.

In consultation with a research librarian, two search strings were created to comprehensively identify publications on IMCI. The first string used the English, French and Spanish names and acronyms of the intervention as text words and adjacent phrases: (“integrated management of childhood illness* (tw)” OR “IMCI (tw)” OR “prise en charge intégrée des maladies de l’enfant* (tw)” OR “PCIME (tw)” OR “Atención integrada a las enfermedades prevalentes de la infancia (tw)” OR “AIEPI (tw)”). The second string sought to identify broader child health interventions that may have adopted components of IMCI as a part of their programs: (“Delivery of Health Care, Integrated (MeSH)” AND (“child health service* (tw)” OR “Child Welfare (MeSH)” OR “child nutrition science* (tw)” OR “child nutrition disorder* (tw)” OR “child* (tw)”)). Terms were truncated to capture alternative spelling and both search queries were linked with the Boolean operator “OR” to expand the search. Because studies of health interventions are often observational in design, no methodological search filters were applied.

### Study Selection

Two of the authors (DTKN & KKL) independently searched and determined the eligibility of the literature by first performing a screen of the titles and abstracts. Abstracts reported in Chinese, French, or Persian were translated by fluent research assistants and other non-English language abstracts were translated using Google Translate software. The initial screening stage was intentionally liberal and all articles reporting original data on IMCI were selected for full-text review. Observed agreement between reviewers at this screening stage was 98.8% (κ = 0.73), and studies rated discordantly were retained for full-text review.

Full-text review was independently performed by the same reviewers for inclusion of the examination of IMCI health worker performance in a primary care setting. Primary care was defined as health facilities that served as the first point of contact for ill children but excluded inpatient hospital settings [24]. Studies were considered eligible if they were randomized controlled trials (RCTs), cluster-RCTs, cohort, pre-post, and cross-sectional studies that included a comparison group of health workers who were unexposed to IMCI training and reported at least one performance outcome of interest: correctly classifying illnesses, vaccinating children with incomplete immunization records, prescribing oral medications, counseling caregivers on nutrition, or instructing caregivers on administering oral therapies. We excluded qualitative studies, case reports, editorials, literature reviews, systematic reviews, as well as studies evaluating the utility of diagnostic algorithms, hospital-based IMCI clinical guidelines, and factors that influence health worker adherence to IMCI protocols. Due to different pathologic and epidemiologic determinants of mortality during the neonatal and perinatal periods [25], studies that consisted exclusively of infants less than two months of age were further excluded. Observed agreement between reviewers for this level of full-text review was 96.1% (κ = 0.87). Disagreements between reviewers were discussed until consensus was reached.

### Data Extraction and Quality Assessment

The reviewers independently extracted the data using an adapted version of the Cochrane Effective Practice and Organization of Care (EPOC) templates for evaluating behavioral interventions [26]. Additional data collected included country, demographic characteristics of health workers and child patients (i.e., sample sizes, age ranges, occupations where applicable), study design, and whether studies were a part of the WHO MCE of IMCI. Among longitudinal studies, the most distal performance evaluation was included in the primary analysis [22], and data from any preceding evaluations were retained for secondary analysis. Potential confounding variables and contributors to heterogeneity were extracted, including the training length, duration between training and performance evaluation, presence of concurrent interventions for child survival, whether health workers received at least one supervisory visit with observed case management in the previous six months, and whether sites had sufficient equipment (≥50% of recommended supplies) for delivering IMCI and vaccination programs [27]. In addition, we documented the presence of additional support and funding to strengthen the IMCI program; where available, we extracted multiple strata from within studies that compared health worker performance under standard IMCI with those receiving additional IMCI supports [28], [29]. We further assessed the influence of baseline health worker performance by dichotomizing studies using the median performance of workers not exposed to IMCI training. To account for broader social determinants of health [30], each study was then linked by country to their corresponding Human Development Index score (HDI) [31]. This composite measure is well-established and is determined based on educational attainment, material wellbeing, and life expectancy [32].

The performance outcomes of interest were developed from the standardized case definitions used in the IMCI Health Facility Survey [25]. Because patients may have multiple concurrent illnesses, the unit of analysis for most studies was the proportion of sick children rather than the proportion of illnesses that were managed correctly. Due to varying endemic diseases across geographic regions [15], we defined the correct prescription of oral medications as providing the necessary antibiotics and/or anti-malarial drugs in the proper formulation and dose. We defined correct nutrition counseling as the proportion of caregivers who were advised to continue feeding and/or provide additional fluids to the sick child. Finally, we defined the correct instruction on administering oral therapies as the proportion of caregivers who were advised on giving the proper dose of necessary antibiotics, anti-malarial drugs, and/or oral rehydration solution.

Lastly, we extracted indicators of study quality according to the recommendations of the Cochrane Handbook [33], [34], and with particular attention to the blinding of outcome assessors, comparability of groups at baseline, and adjustment for confounding [35]. Where possible, the most adjusted relative risks were extracted, although unadjusted relative risks were hand-calculated using the available data in the majority of publications. In instances where insufficient data were available for determining the relative risk or an equivalent point estimate, the corresponding authors were contacted at least twice for supplemental data. If authors were unable to provide the necessary data, the study was excluded from our meta-analysis.

### Data Synthesis and Analysis

Meta-analysis was conducted using Stata version 12 [36], and the “metan” command was used to derive both the pooled relative risks and pooled risk differences. Given the variability in how the interventions were delivered and the diverse health and social contexts across studies, Dersimonian-Laird random-effect models based on the inverse-variance method were used to summarize the effect estimates. This approach produces more conservative pooled estimates and takes into consideration the variation in effect sizes observed between studies [37]. To further contextualize our findings using absolute measures, we calculated the number needed to treat (NNT) for outcomes using the inverse of the pooled risk differences [38].

Because heterogeneity will be present in any public health meta-analysis [23], we addressed this issue in accordance to current recommendations of exploring the underlying variables that drive heterogeneity rather than aborting the analysis [39], [40]. We assessed heterogeneity by visually inspecting the forest plots, and we quantified the magnitude using the I^2^ and Cochran’s Q statistic (significance of *p*<0.05). Galbraith’s plots were then used to qualitatively evaluate the contributions of individual studies to the heterogeneity metrics [41]. Due to the small number of available studies, we did not use multivariate meta-regression which would likely be underpowered [42]. Instead, we performed stratified analyses followed by univariate meta-regression to estimate the amount of heterogeneity attributable to the aforementioned confounding variables [43]. We assessed publication bias using the Begg’s test (significance of *p*<0.05) and visual inspection of the funnel plots.

We further conducted sensitivity analyses restricted to peer-reviewed publications, adjustment for potential confounding, use of blinding, and the comparability of groups at baseline. In eight studies [29], [44], [45], [46], [47], [48], [49], [50], the performance measures were stratified according to the presenting illnesses (e.g., pneumonia, malaria), and analyzed using the proportion of illnesses rather than the proportion of sick children that were correctly classified and managed. To create a single pair-wise comparison for each performance outcome, we combined the illness strata by summing the number of illnesses correctly managed compared with the total number of presenting illnesses encountered by IMCI and non-IMCI health workers, respectively [51]. Recognizing that the subset of patients with multiple concurrent illnesses would contribute to an overestimate of the relative risks, we then performed a sensitivity analysis excluding those studies.

## Results

### Study Selection

**Figure 1:**
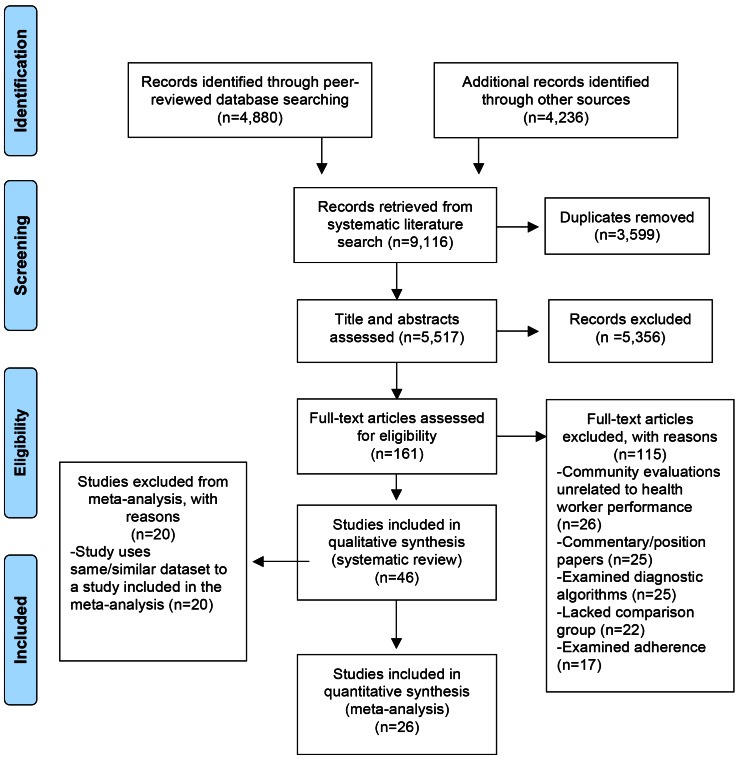
PRISMA flowchart.

The search strategy returned 9,116 citations, of which 4,880 citations were identified from peer-reviewed databases and 4,236 were identified from the grey literature (Figure 1). After excluding 3,599 duplicate citations, the reviewers further excluded 5,356 citations on the basis of the titles and abstracts, leaving 161 articles for full-text review. After reviewing the full-text, 115 articles were excluded for reasons such as lack of comparison groups, commentary papers, investigations of diagnostic algorithms and training adherence. In total, 46 studies were included in the systematic review, of which 26 studies were retained for our meta-analyses.

### Study Characteristics

Characteristics of the included 46 studies are presented in Table S1 [6], [8], [15], [16], [28], [29], [44], [45], [46], [47], [48], [49], [50], [52], [53], [54], [55], [56], [57], [58], [59], [60], [61], [62], [63], [64], [65], [66], [67], [68], [69], [70], [71], [72], [73], [74], [75], [76], [77], [78], [79], [80], [81], [82], [83], [84]. For the meta-analysis, four studies were cluster-RCTs [8], [66], [81], [82], seven studies were pre-post studies [28], [44], [48], [50], [63], [74], [83], and 15 studies were cross-sectional studies [29], [45], [46], [47], [49], [52], [57], [60], [61], [64], [67], [69], [70], [73], [75]. The majority of studies were conducted in Africa [28], [44], [45], [46], [47], [48], [49], [50], [57], [60], [61], [63], [66], [70], [73], [74], [84], followed by Asia [8], [29], [64], [69], [75], [82], [83], and South America [52], [67], [81]. In total, there were 14,582 clinical encounters conducted by health workers (i.e., medical students, medical officers, doctors, health workers, nurses, aides, and lady health visitors) from 1,939 different health facilities. IMCI training ranged from 20 hours to 14 days in duration. Seven studies examined all five performances of interest, including classifying illness, providing vaccinations, prescribing medications, instructing caregivers on administering medications, counseling on nutrition [48], [49], [52], [57], [69], [70], [73]; five studies only examined treatment [47], [60], [64], [74], [83]; two studies only examined a short-course nutrition counseling module [81], [82]; one study only examined caregiver instruction [66]; and 11 studies examined a combination of the four components of health worker performance [8], [28], [29], [44], [45], [46], [50], [61], [63], [67], [75].

Overall, the study quality of the randomized trials was superior to non-randomized studies. Cluster-RCTs tended to report on random sequence generations, attrition rates, and participant exclusions; however, only one study used allocation concealment [8] and two studies used intention to treat analysis [8], [81]. The majority of randomized and non-randomized studies reported taking clustering by facility into consideration, and provided evidence to support the comparability of groups at baseline (Table S1). Few studies blinded the outcome assessors [46], [64], [81], [82].

### Primary Analysis of Health Worker Performance

The health worker performances reported in individual studies are summarized in Table 1, and the pooled effect estimates are presented in Table 2 and graphically in Figures S1 and S2 as a part of our stratified analysis. For the outcomes of illness classification and prescription of medications, the pooled effect estimates suggested that IMCI-trained health workers were more likely to correctly classify illnesses (RR = 1.93, 95% CI: 1.66–2.24) and to prescribe appropriate medications (RR = 1.77, 95% CI: 1.53–2.06) compared with their non-IMCI counterparts (Figures S1a, S1b). However, these findings need to be interpreted with caution given the presence of heterogeneity by both visual inspection and statistical assessment (I^2^ = 78.6% and I^2^ = 94.5% respectively, *p*<0.001). In particular, the Galbraith plots suggested that a cluster-RCT conducted in Bangladesh [8], which reported considerable improvement in care, may have contributed to the heterogeneity. After setting aside this trial, the pooled relative risk for illness classification was comparable to the primary analysis (RR = 1.84, 95% CI: 1.63–2.07) and with reduced heterogeneity (I^2^ = 66.2%, *p*<0.001). No change in the heterogeneity statistic was observed for prescription practices with the exclusion of the trial.

**Table 1:** Percentages of each task performed correctly by IMCI and non-IMCI trained health workers.

Author (Year)	Classification			Medication			Vaccination			Nutrition			Caregiver Instruction		
	IMCI	Non-IMCI	p	IMCI	Non-IMCI	p	IMCI	Non-IMCI	p	IMCI	Non-IMCI	p	IMCI	Non-IMCI	p
	**% (n)**	**% (n)**		**% (n)**	**% (n)**		**% (n)**	**% (n)**		**% (n)**	**% (n)**		**% (n)**	**% (n)**	
Amaral (2004)	65 (294)	31 (358)	<0.001	67 (33)	51 (35)	0.57	39 (96)	38 (105)	0.26	55 (291)	33 (357)	<0.001	57 (68)	25 (102)	0.002
Arifeen (2009)	64 (170)	2 (127)	<0.001	78 (85)	2 (44)	<0.001	–	–	–	67 (169)	0 (123)	<0.001	81 (95)	3 (98)	<0.001
Armstrong (2004)	63 (219)	38 (176)	<0.001	73 (219)	35 (178)	<0.001	12 (69)	0 (27)	>0.10	90 (215)	4 (171)	<0.001	96 (181)	18 (136)	<0.001
Atakouma (2006)	–	–	–	100 (150)	89 (150)	<0.05	–	–	–	–	–	–	–	–	–
Briggs (2002)¥	–	–	–	96 (473)	72 (1066)	<0.001	–	–	–	74 (120)	18 (176)	<0.001	96 (120)	79 (176)	<0.001
Burnham (1997)	–	–	–	42 (126)	35 (97)	0.33	–	–	–	58 (126)	31 (97)	<0.001	57 (126)	42 (97)	–
Choi (2003)¥	60 (132)	23 (143)	<0.001	45 (73)	36 (28)	0.5	93 (214)	84 (146)	0.01	49 (214)	7 (146)	<0.001	44 (214)	19 (146)	<0.001
Chopra (2005)	90 (64)	45 (80)	<0.01	–	–	–	–	–	–	54 (56)	35 (80)	<0.05	–	–	–
Degbey (2005)¥	–	–	–	64 (330)	56 (204)	0.08	–	–	–	–	–	–	–	–	–
Eshaghi (2012)	–	–	–	71 (100)	28 (100)	<0.01	–	–	–	–	–	–	–	–	–
FMOH Sudan (2004)¥	34 (154)	20 (35)	0.16	35 (118)	8 (24)	0.01	–	–	–	42 (269)	0 (81)	<0.001	71 (129)	34 (29)	<0.001
Gilroy (2004)	–	–	–	–	–	–	–	–	–	–	–	–	99 (182)	99 (182)	>0.99
Huicho (2005)	–	–	–	11 (84)	26 (35)	<0.05	5 (38)	7 (15)	0.68	–	–	–	–	–	–
Lee (2001)¥	60 (921)	38 (1462)	<0.001	58 (908)	41 (1447)	<0.001	–	–	–	–	–	–	–	–	–
MOH Viet Nam (2002)	65 (181)	36 (39)	0.001	64 (36)	33 (9)	0.14	5 (39)	0 (9)	0.99	54 (180)	3 (38)	<0.01	64 (66)	13 (15)	<0.001
Naimoli (2006)	68 (242)	51 (225)	<0.001	56 (98)	17 (89)	<0.001	12 (25)	12 (17)	0.99	40 (231)	7 (219)	<0.001	93 (102)	49 (95)	<0.001
Pariyo (2005)	38 (532)	17 (151)	<0.001	54 (414)	40 (108)	<0.05	17 (205)	4 (46)	<0.05	33 (500)	14 (147)	<0.01	68 (476)	31 (134)	<0.001
Rakha (2013)	–	–	–	81 (16)	10 (58)	<0.001	–	–	–	–	–	–	–	–	–
Rehlis (2003)	45 (163)	28 (96)	0.001	–	–	–	–	–	–	61 (163)	50 (96)	0.09	43 (163)	49 (96)	0.37
Rehlis (2007)¥	–	–	–	67 (126)	23 (156)	<0.001	26 (126)	18 (156)	0.11	60 (126)	10 (156)	<0.001	–	–	–
Rowe (2009)	67 (242)	32 (111)	<0.001	54 (184)	10 (79)	<0.001	–	–	–	5 (184)	0 (79)	0.06	–	–	–
Salgado (2002)¥	33 (54)	22 (148)	0.14	50 (18)	43 (42)	0.78	72 (29)	69 (71)	0.81	24 (25)	18 (62)	0.56	35 (23)	23 (62)	0.28
Santos (2001)	–	–	–	–	–	–	–	–	–	63 (166)	59 (175)	<0.001	–	–	–
Uzochukwu (2008)¥	87 (109)	34 (78)	<0.001	82 (44)	26 (31)	<0.001	100 (10)	13 (8)	<0.001	–	–	–	81 (72)	19 (52)	<0.001
Zaman (2008)	–	–	–	–	–	–	–	–	–	33 (52)	4 (53)	0.003	–	–	–
Zhang (2007)	–	–	–	94 (146)	41 (550)	<0.001	–	–	–	–	–	–	–	–	–

–Not reported;

¥sample sizes reflect total illness encounters instead of total child encounters.

**Table 2:** Comparisons of the pooled effect estimates and number needed to treat for each primary outcome and stratified by baseline performance.

Outcome	Pooled Relative Risk		Pooled Risk Difference		Number Needed to Treat	
	RR	(95% CI)	RD (%)	(95% CI) (%)	NNT	(95% CI)
Illness Classification	1.93	(1.66–2.24)	30.1	(22.3–37.9)	4	3–5
Higher Baseline	1.74	(1.52–2.00)	28.5	(14.6–42.4)	4	3–7
Lower Baseline	2.27	(1.66–3.11)	31.1	(22.3–37.9)	4	3–5
Medications	1.77	(1.53–2.06)	29.7	(21.3–38.0)	4	3–5
Higher Baseline	1.42	(1.22–1.64)	19.7	(10.5–28.8)	6	4–10
Lower Baseline	3.08	(2.04–4.66)	41.7	(26.7–56.6)	3	2–4
Vaccinations	1.16	(0.98–1.36)	10.5	(2.6–18.3)	10	6–39
Higher Baseline	1.10	(1.03–1.19)	6.7	(1.9–11.6)	15	7–53
Lower Baseline	3.45	(1.48–8.01)	18.9	(2.6–35.1)	6	3–39
Nutrition	3.57	(2.43–5.25)	33.8	(19.8–47.9)	3	3–5
Higher Baseline	1.74	(1.29–2.35)	21.1	(9.6–32.5)	5	4–11
Lower Baseline	10.12	(6.03–16.99)	45.2	(23.7–66.7)	3	2–5
Caregiver Instruction	2.05	(1.61–2.60)	34.4	(16.3–52.5)	3	2–7
Higher Baseline	1.38	(1.13–1.70)	20.1	(5.2–34.9)	5	3–20
Lower Baseline	3.76	(2.30–6.13)	49.0	(28.6–69.4)	3	2–4

In contrast, the proportions of children requiring immunizations who subsequently received vaccinations from IMCI compared with non-IMCI trained health workers revealed few differences in the presence of broad confidence intervals (Figure S1c). While one small pre-post study conducted in Nigeria [44] reported marked, statistically-significant benefits of training (RR = 5.73, 95% CI: 1.32–24.82), the overall pooled relative risk of 1.16 (95% CI: 0.98–1.36) suggested no difference between groups. However, there was a trend towards statistical significance. There was slight heterogeneity across studies (I^2^ = 22.2%, *p* = 0.24), and visual assessment using the Galbraith plot suggested that this Nigerian study may represent an outlier. When this study was set aside, there was marginal significance with a conservative effect estimate of 1.11 (95% CI: 1.03–1.19, I^2^ = 0%, *p* = 0.56).

With regards to counseling (Figure S2a, S2b), the pooled effect estimates indicated that IMCI health workers were more likely to correctly counsel on basic nutrition (RR = 3.57, 95% CI: 2.43–5.25) and to correctly instruct caregivers on administering oral therapies (RR = 2.05, 95% CI: 1.61–2.60). Although nearly all studies found positive associations between IMCI training and nutrition counseling, visual inspection of the Galbraith plot and statistical assessment for heterogeneity showed much dispersion in the magnitudes of benefit reported by studies (I^2^ = 92.4%, *p*<0.001). Furthermore, this heterogeneity persisted (I^2^ = 91.1%, *p*<0.001) even after excluding two cluster-RCTs [81], [82] that examined a short-course nutrition module (RR = 3.85, 95% CI: 2.54–5.83). Likewise, a similar degree of heterogeneity was observed among studies that examined counseling on administering oral therapies (I^2^ = 95.3%, *p*<0.001).

### Stratified Analysis

**Figure 2:**
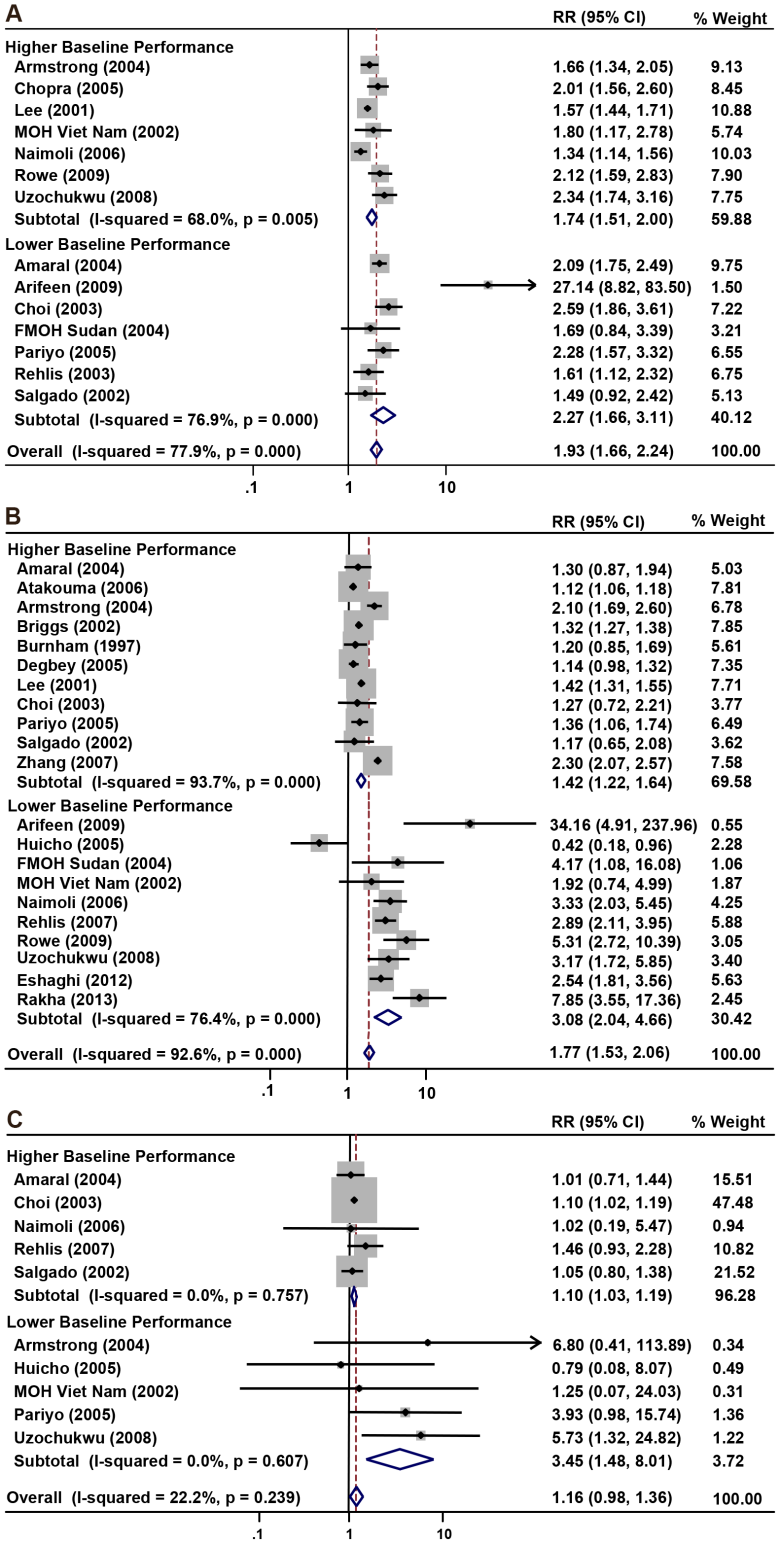
Forest plots showing pooled point estimates for various outcomes stratified by baseline performance. (a) Illness classification stratified by baseline performance. (b) Medications stratified by baseline performance. (c) Vaccinations stratified by baseline performance.

**Figure 3:**
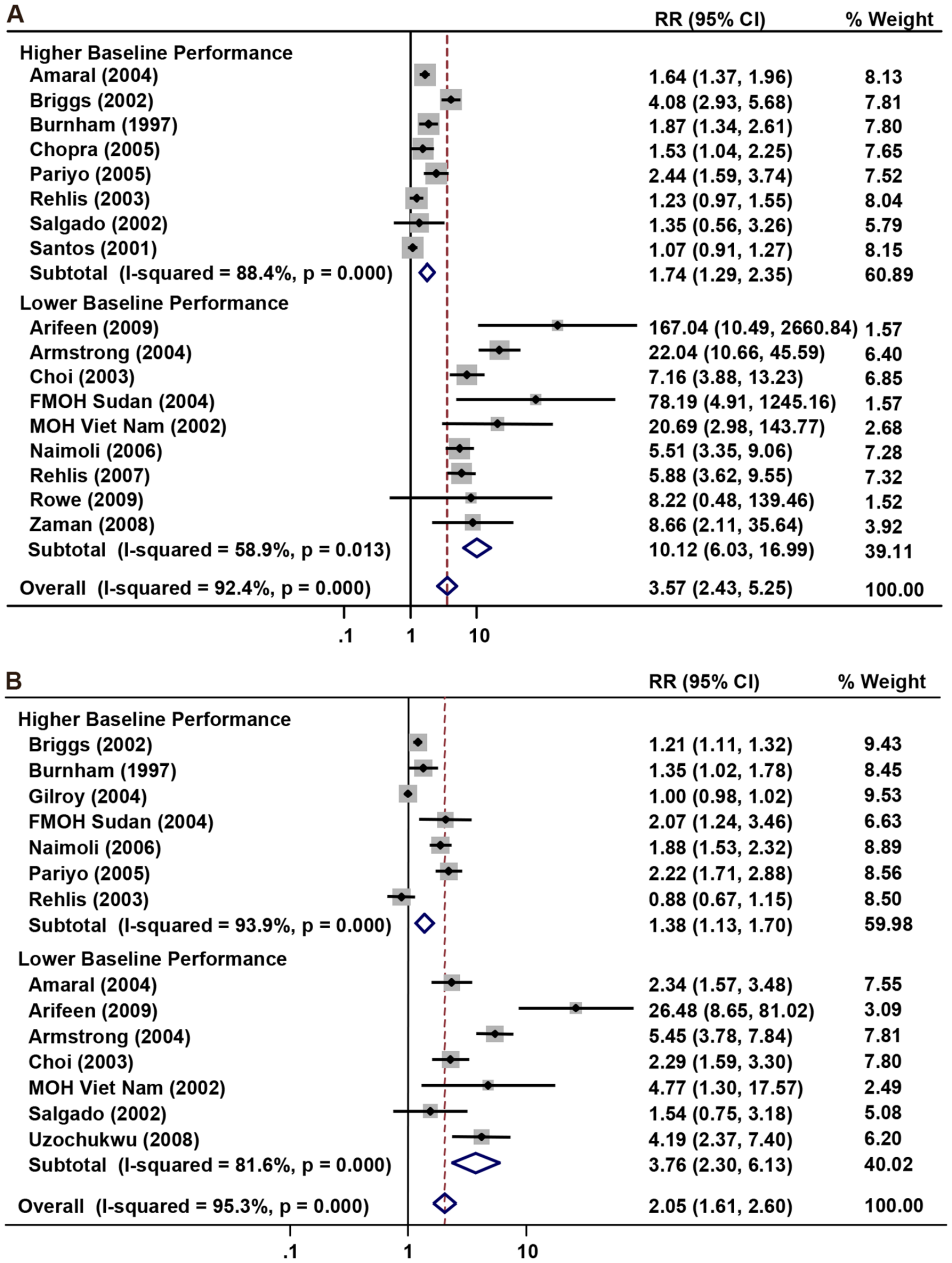
Forest plots showing pooled point estimates for counseling outcomes stratified by baseline performance. (a) Nutrition stratified by baseline performance. (b) Instruction stratified by baseline performance.

To explore the heterogeneity observed in the primary analyses, we conducted subgroup analyses for each performance domain (Table 3). Using the median values of health workers unexposed to IMCI as an index of baseline performance, greater benefits of IMCI training were evident in most domains among studies with lower performances at baseline (Figures 2, 3). In particular, there were approximately three-fold increases in correctly prescribing medications, providing vaccinations, and counseling on oral therapies, with no overlap in their respective confidence intervals. Significant benefit was seen in nutrition counseling as well (RR = 10.12, 95% CI: 6.03–16.99). Using univariate analysis, baseline performance accounted for 9.2% of the heterogeneity in prescribing medications, 44.2% in vaccinations, 61.9% in nutrition counseling, 16.3% in counseling on oral therapies, but was not associated with classifying illnesses. Similar patterns were observed in the analyses by HDI, with stronger, significant associations seen in lower resource settings for nutrition counseling (RR = 5.11, 95% CI: 2.95–8.87) and a trend towards significance for vaccinations (RR = 1.27, 95% CI: 0.98–1.63). Notably, HDI accounted for all of the heterogeneity in vaccinations, which likely reflects a correlation between national wealth and health worker performance at baseline. Moreover, while stratification by study design showed greater effects of cluster-RCTs, this trend was primarily driven by one trial [8]. Concordance was generally evident in the results of cross-sectional and pre-post studies (Figures S1, S2), with study design accounting for 22.2% of the heterogeneity in illness classification.

**Table 3:** Stratified analyses of IMCI and non-IMCI trained health workers’ performance outcomes with the number of pooled studies before each point estimate.

Indicator	Classification			Medication			Vaccination			Nutrition			Caregiver Instruction		
	No.	RR (95% CI)	I^2^	No.	RR (95% CI)	I^2^	No.	RR (95% CI)	I^2^	No.	RR (95% CI)	I^2^	No.	RR (95% CI)	I^2^
**Baseline Performance**															
Higher	7	1.74 (1.52–2.00)	68	11	1.42 (1.22–1.64)	94	5	1.10 (1.03–1.19)	0	8	1.74 (1.29–2.35)	88	7	1.38 (1.13–1.70)	94
Lower	7	2.27 (1.66–3.11)	77	10	3.08 (2.04–4.66)	76	5	3.45 (1.49–8.01)	0	6	10.12 (6.03–16.99)	59	7	3.76 (2.30–6.13)	82
**HDI**															
Higher	3	1.77 (1.29–2.41)	88	5	2.04 (1.16–3.59)	89	3	1.01 (0.72–1.41)	0	4	1.87 (1.16–3.02)	93	2	1.97 (1.64–2.38)	0
Lower	11	2.02 (1.67–2.44)	77	16	1.63 (1.42–1.87)	89	7	1.27 (0.98–1.63)	46	13	5.11 (2.95–8.87)	91	12	2.03 (1.57–2.62)	95
**Study Design**															
Cross–Sectional	8	1.71 (1.46–2.02)	59	14	1.53 (1.32–1.78)	89	8	1.14 (0.94–1.38)	0	11	3.60 (2.28–5.69)	92	10	1.89 (1.39–2.57)	92
Pre–Post Study	5	2.04 (1.64–2.54)	78	6	2.53 (1.74–3.67)	94	2	2.13 (0.44–10.29)	79	3	3.73 (0.97–14.40)	89	2	2.97 (1.66–5.34)	67
Cluster RCT	1	27.14 (8.82–83.51)	–	1	34.17 (4.91–237.96)	–	–	–	–	3	8.27 (0.72–94.85)	90	2	4.90 (0.20–121.23)	97
**MCE Studies**															
Yes	4	2.61 (1.68–4.04)	88	5	1.49 (0.91–2.44)	86	4	1.62 (0.66–4.00)	41	4	6.38 (2.01–20.25)	95	4	4.34 (2.21–8.54)	91
No	10	1.80 (1.56–2.08)	66	16	1.85 (1.57–2.19)	94	6	1.15 (0.97–1.36)	23	13	3.23 (2.04–5.09)	92	10	1.54 (1.25–1.89)	92
**Training Length**															
≥11 Days	9	1.96 (1.60–2.39)	76	7	2.15 (1.54–3.02)	84	3	3.62 (1.15–11.41)	0	8	6.05 (2.61–14.02)	92	7	2.63 (1.48–4.69)	96
<11 Days	5	1.91 (1.44–2.52)	84	14	1.69 (1.40–2.03)	94	7	1.12 (0.99–1.27)	12	9	3.00 (1.88–4.80)	94	7	1.87 (1.40–2.50)	87
**Duration since Training**															
≥1 Year	2	1.80 (1.49–2.18)	25	2	2.26 (2.05–2.49)	0	1	6.80 (0.41–113.89)	–	2	5.70 (0.42–77.82)	98	1	5.45 (3.79–7.84)	–
<1 Year	12	1.97 (1.65–2.35)	81	19	1.66 (1.44–1.90)	89	9	1.14 (0.98–1.33)	20	15	3.27 (2.22–4.82)	91	13	1.83 (1.47–2.28)	94
**Supervisory Visits**															
≥1 in 6 Months	6	2.09 (1.57–2.78)	86	8	1.91 (1.31–2.80)	89	5	1.11 (1.03–1.20)	0	6	5.97 (2.26–15.75)	92	5	3.18 (1.28–7.88)	97
<1 in 6 Months	8	1.88 (1.56–2.26)	69	13	1.73 (1.44–2.07)	94	5	1.84 (0.81–4.19)	51	11	2.64 (1.79–3.89)	91	9	1.79 (1.37–2.35)	88
**Sufficient Equipment**															
≥50% Supplies	7	1.84 (1.52–2.23)	74	9	1.84 (1.38–2.47)	85	7	1.10 (1.02–1.19)	0	7	4.21 (2.26–7.86)	94	7	2.27 (1.48–3.49)	91
<50% Supplies	7	2.13 (1.60–2.82)	83	12	1.62 (1.39–1.88)	90	3	1.44 (0.84–2.44)	67	10	3.28 (1.85–5.81)	91	7	1.67 (1.30–2.15)	93
**Additional Supports**															
Yes	5	2.02 (1.49–2.75)	86	6	2.30 (1.60–3.30)	90	2	1.64 (0.74–3.61)	11	6	5.66 (2.43–13.21)	93	4	2.99 (1.02–8.76)	97
No/Not Reported	9	1.93 (1.61–2.32)	70	15	1.59 (1.32–1.92)	94	8	1.12 (0.95–1.31)	18	11	3.01 (2.00–4.63)	91	10	2.05 (1.57–2.68)	96
**Concurrent Interventions**															
Yes	7	2.15 (1.65–2.80)	80	9	1.70 (1.25–2.32)	94	5	1.10 (1.02–1.18)	0	8	4.08 (2.15–7.74)	94	7	2.43 (1.44–4.11)	95
No/Not Reported	7	1.77 (1.50–2.08)	68	12	1.85 (1.51–2.26)	91	5	1.92 (1.09–3.41)	18	9	3.37 (1.94–5.85)	92	7	1.95 (1.31–2.92)	95
**Sensitivity Analyses**															
Adjusted Analyses	8	1.91 (1.56–2.34)	84	8	2.15 (1.58–2.92)	85	4	1.57 (0.69–3.54)	41	9	4.90 (2.61–9.23)	94	7	2.75 (1.60–4.73)	97
Blinding	1	1.69 (0.84–3.40)	–	2	2.61 (1.88–3.63)	0	–	–	–	3	6.46 (0.69–60.16)	88	1	2.07 (1.24–3.46)	–
Comparable Baseline	10	2.03 (1.70–2.43)	85	12	2.10 (1.65–2.68)	92	6	1.30 (0.90–1.87)	49	10	3.99 (2.33–6.83)	94	8	2.93 (1.75–4.91)	97
Excl. Grey Lit	8	2.11 (1.67–2.67)	85	12	2.23 (1.61–3.08)	95	6	1.90 (0.86–4.18)	48	9	3.61 (2.10–6.22)	93	7	3.06 (1.74–5.38)	97
Excl. Composite	10	1.96 (1.59–2.40)	79	15	2.02 (1.51–2.70)	94	8	1.14 (0.94–1.38)	0	16	3.35 (2.27–4.94)	92	11	1.93 (1.50–2.49)	96

Abbreviations: RR = Pooled relative risk; 95% CI = 95% confidence intervals; HDI = Human Development Index; MCE = Multi-Country Evaluation; C-RCT = cluster randomized controlled trial, Excl. Grey Lit = excluding grey literature; Excl. Composite = excluding composite proportions based on illness encounters instead of child encounters; –Not reported.

Among confounding factors pertaining to the intervention design, the following trends require conservative interpretation given the broad and frequently overlapping confidence intervals between strata. Stronger associations were observed among studies reporting more supervisory visits for most domains including vaccinations (RR = 1.11, 95% CI: 1.03–1.20), although this variable accounted for less than 5.5% of the heterogeneity in all outcomes. Likewise, a stronger performance was seen in vaccinations among studies describing sufficient medical equipment for IMCI (RR = 1.10, 95% CI: 1.02–1.19), and a longer training duration (RR = 3.62, 95% CI: 1.15–11.41). Additional supports and funding to strengthen IMCI was similarly associated with improved performance in most domains (Table 3). However, because of the paucity of studies reporting on study quality indicators, the sensitivity analyses revealed no clear trends regarding the adjustment for confounding, comparability at baseline, and blinding.

### Measures of Absolute Effects

To contextualize these findings, the pooled risk differences and NNTs were determined for each outcome and baseline performance strata (Table 2). The absolute effect estimates were in favor of IMCI training, with a 30.1% difference in classifying illnesses. Among studies with a lower baseline performance, training was associated with a 42.7% difference in prescribing appropriate medications, 18.9% difference in providing vaccinations, 45.2% difference in counseling on nutrition, and 49.0% difference in instructing caregivers on administering oral therapies. The corresponding NNTs suggested that providing IMCI-informed care to four children is needed to enable the correct illness classification for one additional child, six children to enable vaccination provision for one additional child, and three children to respectively enable the medication prescription, correct nutrition counseling, or caregiver instruction for one additional child.

### Publication Bias

Inspection of the funnel plots showed general symmetry and little evidence for publication bias. The Begg’s tests approached statistical significance for illness classification (*p* = 0.05), but were not statistically significant for medication prescription (*p* = 0.24), vaccinations (*p* = 0.37), nutrition counseling (*p* = 0.09), and caregiver instruction on oral therapies (*p* = 0.23).

## Discussion

With less than three years remaining of the fourth Millennium Development Goal of reducing child mortality by two-thirds [85], few countries are currently projected to achieve the necessary gains required for child survival [2], [86]. Sustained reductions in child mortality and morbidity necessitate adopting a multipronged approach including community health promotion and strengthening service provision [87], of which mobilizing human resources and skills are core components [88], [89]. In this systematic review of 46 studies, IMCI training was associated with significant improvements in quality of care, and this relationship was further modified by health worker performance at baseline. Although our findings need to be interpreted cautiously in the presence of heterogeneity and the limitations inherent to observational studies, greater gains were evident in prescribing medications, vaccinating children, counseling on adequate nutrition, and instructing caregivers on administering oral therapies among studies of health workers with lower baseline performance. IMCI health workers were also more likely than their non-IMCI counterparts to correctly classify illnesses regardless of baseline performance.

Less benefit was observed in the pooled vaccination rates compared to other outcomes, which is notable given the epidemiological evidence and historical precedence supporting its efficacy in reducing mortality [90], [91]. Our analysis may be potentially underpowered to detect differences due to the relatively small pooled sample of 600 cases. However, this finding may reflect important residual confounding. While most studies reported sufficient materials to support immunizations [52], and indeed our analysis suggested that adequate equipment modestly improves rates, other contextual factors may have influenced uptake including parallel vaccination programs, cultural acceptability of vaccinating presently-ill children [92], and geographical barriers that impede access to preventive care [67]. Moreover, while countries such as Tanzania and Uganda had policies of opening a vaccine vial even for one child with an incomplete vaccination record, the poor cost-effectiveness often resulted in deferral until scheduled community-wide vaccinations [57], [73], [81]. Because of the cross-sectional nature of the studies, we were unable to ascertain the proportion of children who ultimately received vaccinations at follow-up. Thus, we cannot exclude the possibility of incomplete capture rates of this outcome, which may contribute to bias towards the null.

Despite statistically-significant improvements in clinical skills, we caution readers that the absolute proportions of children who received appropriate care was often low [73]. In 13 of the 21 studies that reported on prescription practices, at least one-third of the children seen by IMCI health workers were prescribed incorrect medications. This finding is concerning, as providing antibiotics for pneumonia and sepsis alone could reduce the global child mortality rate by 12% [19]. Factors related to the implementation of IMCI may account for these trends [93]. First, low coverage rates may have diluted the effects of the intervention, as facilities often consisted of both IMCI and non-IMCI health workers because of poor staff retention and high training costs [46], [52], [94]. The delivery of the intervention was also variable in terms of the duration of training. Our results are consistent with a previous review [22], and suggest that a longer training duration may be associated with improved performance. Furthermore, studies have found that while training improves knowledge on written exams, scores were rarely superior in performance [95], which raises concerns regarding how much knowledge is actually retained, and thus, translatable into practice.

Overall, these findings are consistent with previous research, where continued medical education is associated with moderate effect sizes for improving health provider behaviors [22], [96]. In particular, our stratified analysis suggest that IMCI training may produce greater gains in settings with lower health worker performance and fewer HDI-measured resources, such as in Asia and Sub-Saharan Africa – regions that continue to bear most of the child mortality burden [86], [97]. Extensive literature further show that strengthening health worker performance is multifactorial and reflects dynamic interactions with health system factors including the frequency and quality of supervision [22], [89], health worker motivation [88], presence of additional funding and partnerships to sustain programs [8], [98], and investment in infrastructure [16], [99]. Our stratified analysis lends support that greater supervision with case management observation, additional resources to strengthen IMCI, and equipment adequacy enhances performance especially in the counseling domains.

### Strengths and Limitations

The strengths of our review include using a predetermined protocol, comprehensive searches without language restrictions of both the peer-reviewed and grey literature, and standardized outcomes based on the WHO Health Facility Survey which enabled cross-study comparisons [25]. While there was a trend toward publication bias in one outcome, we attempted to ameliorate this issue by contacting other research teams regarding additional or ongoing studies. Because methodological plurality is common in public health interventions, we also evaluated the quality of both randomized and non-randomized studies with specific attention to randomization, comparability of groups, confounding, and blinding, and assessed the effects of selection and detection bias using stratified analysis [33], [34], [100].

This review also has limitations and methodological considerations. First, IMCI has been introduced in over 100 countries [101], but only 45 studies met our inclusion criteria and 26 studies contributed data to the meta-analysis. These findings reflect a subset of countries that have adopted and studied this strategy, which may limit generalizability to other contexts. Second, most of the included studies were observational in design, which precludes causal conclusions. We further pooled the findings from different study designs together only after stratification revealed general concordance in the results except for one cluster-RCT [8]. Third, despite sizable heterogeneity, we have quantitatively synthesized the effects of IMCI training. Heterogeneity is an expectation rather than the exception in public health meta-analyses [102], [103]. To increase the transparency in our approach to heterogeneity, we have presented a sequential analysis including using Galbraith plots to assess for outlying studies, conducting exploratory stratified analyses of established confounding variables, and triangulating with univariate meta-regression to estimate the amount of attributable variance. While heterogeneity can reduce the interpretability of results [104], we note that most studies reported positive associations, and the varying magnitude of benefits may be accounted for by differing baseline performances [105].

Fourth, as best as possible, we extracted key contextual factors that help illustrate the complexity of these studies including the availability of resources to strengthen IMCI, and presence of concurrent interventions such as insecticide-treated nets (ITN), parallel vaccination programs, and vitamin supplementation [99]. Our analysis of concurrent interventions, for instance, failed to detect any differences in performance. This result may reflect misclassification bias, as lack of reporting within studies does not necessarily equate to their absence, and would have contributed bias toward the null. Alternatively, it is possible that interventions such as ITN distribution would have limited impact on health worker performance in unrelated skills such as diagnosis and nutrition counseling. Fifth, due to our small sample of studies, we could not assess the interactions between multiple confounders. For example, the Bangladeshi cluster-RCT [8] had a lower HDI, poorer baseline health worker performance, and was unique because it sought to determine the efficacy of IMCI training under optimal conditions, including implementing all three IMCI components, attaining 90% supervisory rates, and achieving governmental, religious, and community support of IMCI [55]. Therefore, the complexity of public health studies warrants care when considering confounding variables in isolation.

Sixth, other variables that we could not assess included the contamination effects of staff turnover and transfers between facilities. However, our secondary analyses comparing successive health worker cohorts within studies did not reveal any significant differences between groups (results not presented). Furthermore, we were unable to assess the influences of pre-service training [106] and the IMCI community component on health worker performance. Seventh, while most studies reported accounting for clustering, few provided the intraclass-correlation coefficients for facilities and children seen by the same health worker [8], [66]. We were unable to appropriately adjust for these correlations, which may have resulted in an overestimation of the relative risks and their precision [107].

### Future Directions

Public health interventions by nature are complex, situation-dependent, and programmatic, as evident in the case of IMCI where countries have adapted this strategy according to their epidemiological profile and available resources [67]. In this review, we have primarily adopted an epidemiological approach to quantifying the effectiveness of IMCI training. In order to strengthen IMCI and the development of worker competencies, however, an analysis of the sociocultural and contextual determinants of performance is need to identify the specific factors that influence, support, and hinder adherence to evidence-based care [15], [98]. A realist review may be a suitable framework for guiding a detailed, explanatory analysis into for whom this intervention benefits, the circumstances in which it thrives, the available resources and presence of community and government buy-in, and the components of the intervention that require modification to enhance effectiveness [108]. Because a realist review seeks to synthesize a broad range of evidence including qualitative research and case reports [109], this method could comprehensively evaluate the literature that did not meet our inclusion criteria, but nonetheless provide rich, nuanced insights into health worker performance.

Ultimately, whether IMCI reduces child mortality remains unclear [8], [57], [67], [74], [79], [110], [111], and to our knowledge, a systematic review of the neonatal component of IMCI (IMNCI) has yet to be undertaken, even though neonatal deaths account for over two-fifths of the under-five mortality [86]. Different baseline mortality rates, diverse methods for measuring this parameter [8], and variability in implementation have contributed to this lack of clarity [98]. Inconsistent reporting of non-randomized studies further impede the appraisal process, and adopting standardized reporting procedures, such as TREND, is essential for improving evaluations of public health research [112]. With emergent techniques for estimating the impacts of scaling up interventions [113], [114], additional RCTs and quality longitudinal studies are needed not only for understanding the mechanisms that mediate improvements in pediatric care, but also for determining the survival benefits that are likely obtainable.

## Acknowledgments

We thank Diane Lorenzetti from the University of Calgary for her invaluable assistance in the design of our literature search strategy. We thank Drs. Lulu Muhe and Thierry Lambrechts from the World Health Organization and Drs. Samantha Y. Rowe and Alexander K. Rowe from the Malaria Branch, Division of Parasitic Diseases and Malaria, Center for Global Health, Centers for Disease Control and Prevention, Atlanta, Georgia, for allowing us access to their literature database. Special thanks to Dr. A.K. Rowe and our reviewers for their insightful comments during the refinement of this manuscript.

## Supporting Information

Figure S1 **Forest plots showing pooled point estimates for various outcomes stratified by study design.** (a) Illness classification stratified by study design. (b) Medications stratified by study design. (c) Vaccinations stratified by study design.
(TIF)

Figure S2 **Forest plots showing pooled point estimates for counseling outcomes stratified by study design.** (a) Nutrition stratified by study design. (b) Instruction stratified by study design.
(TIF)

Table S1 **Characteristics of studies comparing Integrated Management of Childhood Illness (IMCI) trained and non-IMCI trained health workers.** Abbreviations: ¥ = Indicates Multi-Country Evaluation Study; HDI = Human Development Index; Med = Medium human development index; n.r. = Not reported; mo = months; Suff. Equip. = Sufficient equipment required for delivering IMCI; Suff. Vacc. = Sufficient supplies to deliver vaccination programs; Adj. RR = Adjusted relative risk; Pats. = patients; prg = program; yr = years Training length = Number of days workers were trained in IMCI; Durations since Training = Time from completion of IMCI training and assessment of worker performance; Supervisory visits = Health workers received at least one supervisory visit with observed case management in the previous six month
(DOC)

Text S1 **Protocol (version 4.3).** Last updated on November 28^th^, 2012.
(DOC)

Text S2 **PRISMA checklist.**
(DOC)
