# Supplementary figures and images for: Does Integrated Management of Childhood Illness (IMCI) Training Improve the Skills of Health Workers? A Systematic Review and Meta-Analysis
Source: PLoS One. 2013 Jun 12;8(6):e66030. doi: 10.1371/journal.pone.0066030 (PMC3680429; doi:10.1371/journal.pone.0066030)

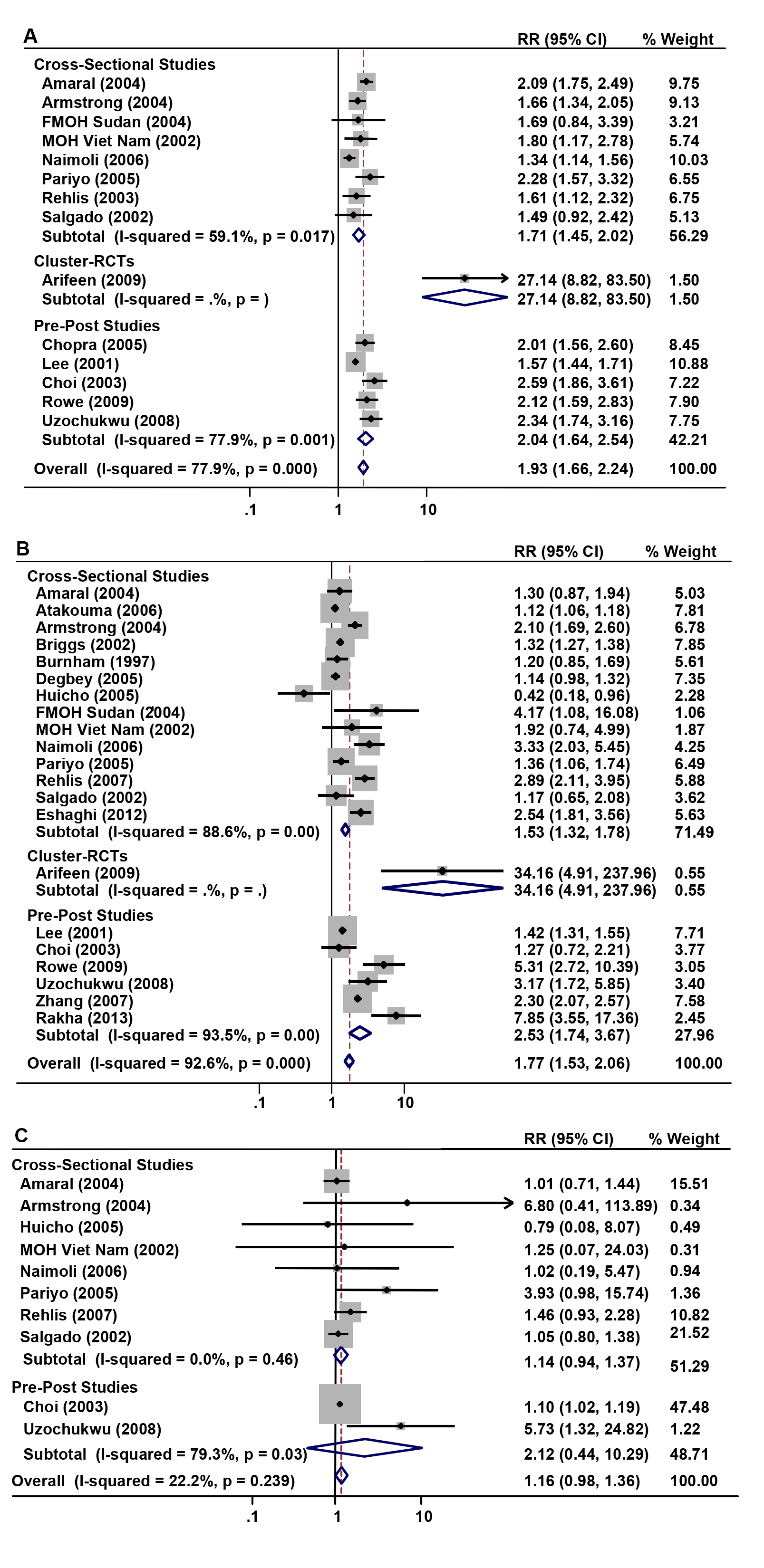

Supplement: Figure S1 — Forest plots showing pooled point estimates for various outcomes stratified by study design. (a) Illness classification stratified by study design. (b) Medications stratified by study design. (c) Vaccinations stratified by study design. (TIF) [file pone.0066030.s001.tif]

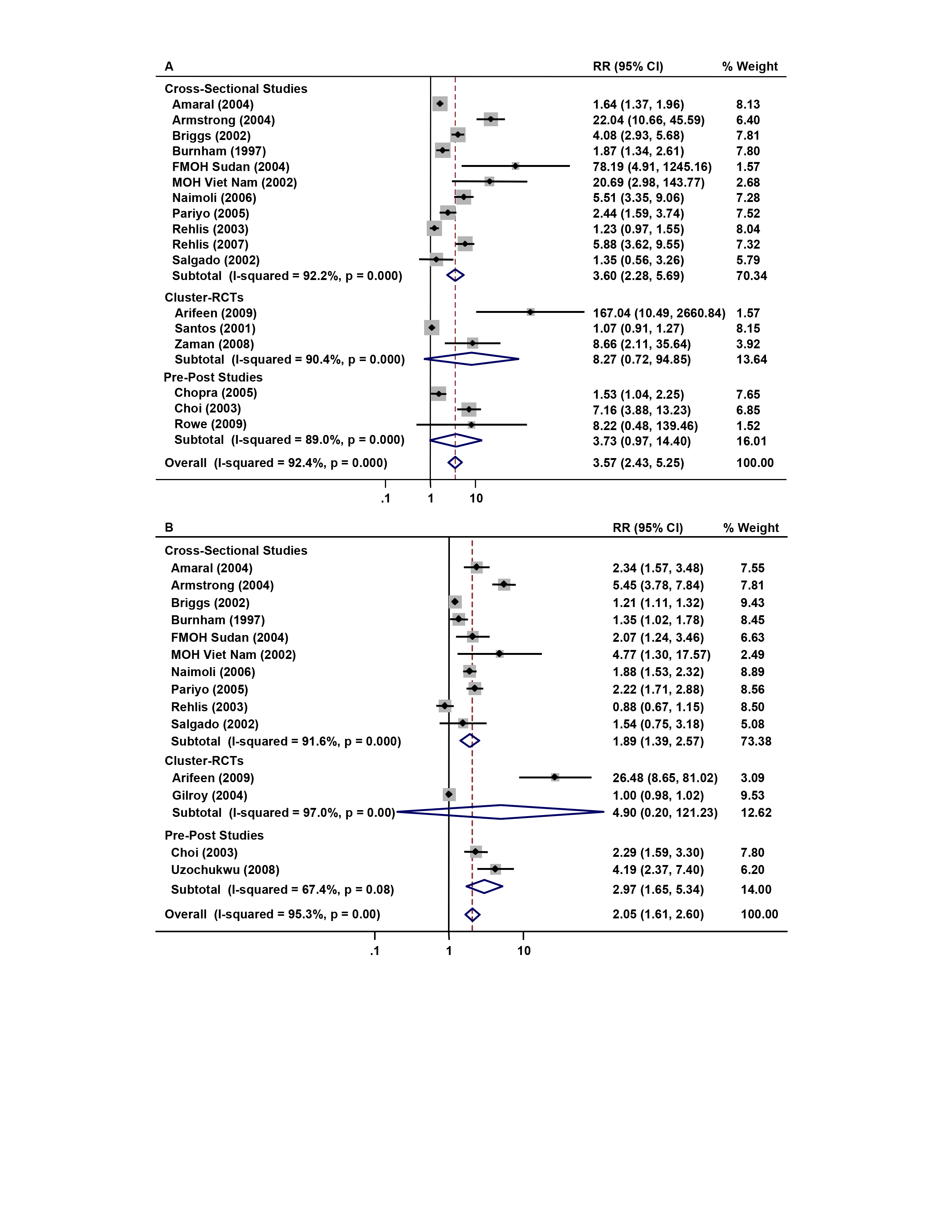

Supplement: Figure S2 — Forest plots showing pooled point estimates for counseling outcomes stratified by study design. (a) Nutrition stratified by study design. (b) Instruction stratified by study design. (TIF) [file pone.0066030.s002.tif]
