# Supplementary material for: Does Integrated Management of Childhood Illness (IMCI) Training Improve the Skills of Health Workers? A Systematic Review and Meta-Analysis
Source: PLoS One. 2013 Jun 12;8(6):e66030. doi: 10.1371/journal.pone.0066030 (PMC3680429; doi:10.1371/journal.pone.0066030)
Supplement: Table S1 — Characteristics of studies comparing Integrated Management of Childhood Illness (IMCI) trained and non-IMCI trained health workers. Abbreviations: ¥ = Indicates Multi-Country Evaluation Study; HDI = Human Development Index; Med = Medium human development index; n.r. = Not reported; mo = months; Suff. Equip. = Sufficient equipment required for delivering IMCI; Suff. Vacc. = Sufficient supplies to deliver vaccination programs; Adj. RR = Adjusted relative risk; Pats. = patients; prg = program; yr = years Training length = Number of days workers were trained in IMCI; Durations since Training = Time from completion of IMCI training and assessment of worker performance; Supervisory visits = Health workers received at least one supervisory visit with observed case management in the previous six month (DOC) [file pone.0066030.s003.doc]

Table S1. Characteristics of studies comparing Integrated Management of Childhood Illness (IMCI) trained and non-IMCI trained health workers.

| **Studies (1st study is meta-paper)** | **Country** | **HDI** | **Sample size** | | | **Training length (days)** | **Duration since training (≥ 1 yr)** | **Sup. visit (≥ 1 in 6 mo)** | **Suff. equip. (≥ 50%)** | **Suffi. vacc. (≥ 50%)** | **Concurrent intervention(s)** | **Study quality indicators** | | |
| --- | --- | --- | --- | --- | --- | --- | --- | --- | --- | --- | --- | --- | --- | --- |
|  |  |  | **# Health Facilities** | **# Health Workers** | **#Pats. (<5 yr)** |  |  |  |  |  |  | **Blinding outcome assessor** | **Comparable baseline** | **Adjusted RR analysis** |
| Amaral et al., 2004¥; Gouws et al., 2004 ; Amaral, 2002 | Brazil | High | 24 | 653 | 653 | 6 to 8 | 0 | 0 | Yes | Yes | Family health program; Community health worker's program | 0 | Yes | Yes |
| Arifeen et al., 2009¥ ; Arifeen et al., 2005 ; Arifeen et al., 2004 ; Arifeen, 2000 | Bangladesh | Low | 20 | n.r. | 539 | 2 to 15 | 0 | Yes | 0 | 0 | Family planning services; Vitamin A supplementation; Immunization; national nutrition programme | 0 | Yes | Yes |
| Armstrong, Bryce, et al., 2004¥ ; Bryce et al., 2005; Armstrong, Adam, et al., 2004 ; Gouws et al., 2004 ; Mgalula, 2000 ; Mbuya et al., 2003 | Tanzania | Low | 73 | n.r. | 419 | 11 | Yes | Yes | Yes | Yes | Insecticide-treated nets | 0 | Yes | Yes |
| Atakouma et al., 2006 | Togo | Low | n.r. | n.r. | 300 | n.r. | 0 | 0 | 0 | 0 |  | 0 | 0 | 0 |
| Briggs et al., 2002 | Senegal | Low | 41 | n.r. | 3,033 | n.r. | 0 | 0 | 0 | 0 | Drug kits; Bamako initiative; | 0 | 0 | 0 |
| Burnham, 1997 | Zambia | Low | 8 | n.r. | 223 | n.r. | 0 | 0 | 0 | 0 |  | 0 | 0 | 0 |
| Choi et al., 2003 ; Mehari et al., 2000 | Eritrea | Low | 75 | n.r. | 360 | n.r. | 0 | Yes | Yes | Yes | Malaria program | 0 | Yes | 0 |

Abbreviations: ¥ = Indicates Multi-Country Evaluation Study; HDI = Human Development Index; Med =Medium human development index; n.r. = Not reported; mo = months; Suff. Equip. = Sufficient equipment required for delivering IMCI; Suff. Vacc. = Sufficient supplies to deliver vaccination programs; Adj. RR = Adjusted relative risk; Pats. = patients; prg = program; yr = years Training length = Number of days workers were trained in IMCI; Durations since Training = Time from completion of IMCI training and assessment of worker performance; Supervisory visits = Health workers received at least one supervisory visit with observed case management in the previous six month

Table S1: continued

| **Studies (1st study is meta-paper)** | **Country** | **HDI** | **Sample size** | | | **Training length (days)** | **Duration since training (≥ 1 yr)** | **Sup. visit (≥ 1 in 6 mo)** | **Suff. equip. (≥ 50%)** | **Suffi. vacc. (≥ 50%)** | **Concurrent intervention(s)** | **Study quality indicators** | | |
| --- | --- | --- | --- | --- | --- | --- | --- | --- | --- | --- | --- | --- | --- | --- |
|  |  |  | **# Health Facilities** | **# Health Workers** | **# Pats (<5 yr)** |  |  |  |  |  |  | **Blinding outcome assessor** | **Comparable baseline** | **Adjusted RR analysis** |
| Chopra et al., 2005 | South Africa | Med | 21 | 21 | 160 | 11 | Yes | Yes | 0 | 0 |  | 0 | Yes | 0 |
| Degbey, 2005 | Niger | Low | 44 | n.r. | 267 | n.r. | 0 | 0 | 0 | Yes |  | 0 | Yes | 0 |
| Eshaghi et al., 2012 | Iran | High | 2 | 200 | 200 | 1 | 0 | 0 | 0 | 0 |  | Yes | 0 | 0 |
| FMOH Sudan, 2004 ; WHO EMRO 2004 | Sudan | Low | 66 | n.r. | 364 | 11 | 0 | 0 | 0 | 0 |  | Yes | 0 | Yes |
| Gilroy et al., 2004 | Mali | Low | 10 | 10 | 364 | 11 | 0 | Yes | 0 | 0 |  | 0 | Yes | Yes |
| Huicho et al., 2005¥ ; MOH Peru, 1999 | Peru | High | 90 | 202 | 428 | 7 | 0 | 0 | Yes | Yes | Expanded program on immunization; Growth & development program; Program for the prevention of minerals & micronutrient deficiencies | 0 | 0 | 0 |
| Lee et al., 2001 | Kenya | Low | 36 | n.r. | 1,333 | 15 | 0 | Yes | 0 | 0 |  | 0 | Yes | Yes |
| MOH Viet Nam, 2002 | Viet Nam | Med | 70 | n.r. | 220 | 11 | 0 | 0 | Yes | Yes |  | 0 | 0 | 0 |
| Naimoli et al., 2006 ; Naimoli, 2001 ; 2000 | Morocco | Med | 62 | 101 | 467 | 12 | 0 | 0 | Yes | Yes |  | 0 | Yes | Yes |
| Pariyo et al., 2005¥ ; Gouws et al., 2004 | Uganda | Low | 316 | 427 | 1534 | 11 to 14 | 0 | 0 | Yes | 0 |  | 0 | Yes | Yes |
| Rakha et al., 2013 ; El Mahalli & Aki, 2011 | Egypt | Med | 97 | n.r. | 74 | 4 to 7 | 0 | 1 | 1 | 1 |  | 0 | Yes | 0 |

Table S1: continued

| **Studies (1st study is meta-paper)** | **Country** | **HDI** | **Sample size** | | | **Training length (days)** | **Duration since training (≥ 1 yr)** | **Sup. visit (≥ 1 in 6 mo)** | **Suff. equip. (≥ 50%)** | **Suffi. vacc. (≥ 50%)** | **Concurrent intervention(s)** | **Study quality indicators** | | |
| --- | --- | --- | --- | --- | --- | --- | --- | --- | --- | --- | --- | --- | --- | --- |
|  |  |  | **# Health Facilities** | **# Health Workers** | **# Pats (<5 yr)** |  |  |  |  |  |  | **Blinding outcome assessor** | **Comparable baseline** | **Adjusted RR analysis** |
| Rehlis, 2003 | Uzbekistan | Med | 120 | 179 | 170 | 11 | 0 | 0 | Yes | Yes | Health-1 Project | 0 | 0 | 0 |
| Rehlis, 2007 ; Rathmony, 2006a ; 2006b | Cambodia | Med | 120 | n.r. | 456 | n.r. | 0 | Yes | 0 | Yes |  | 0 | 0 | 0 |
| Rowe et al., 2009 ; Rowe, Osterholt et al., 2012 ; Rowe et al., 2011 ; Osterholt et al., 2009 | Benin | Low | 114 | 267 | 1,244 | 11 | 0 | 0 | 0 | 0 | Africa Integrated Malaria Initiative | 0 | Yes | 0 |
| Salgado et al., 2002 | Ethiopia | Low | 43 | 43 | 201 | n.r. | 0 | Yes | 0 | Yes | Essential services for health in Ethiopia; BASICS project | 0 | 0 | 0 |
| Santos et al., 2001 | Brazil | High | 28 | 33 | 424 | 20 hours | 0 | 0 | 0 | 0 |  | Yes | Yes | Yes |
| Uzochukwu et al., 2008 | Nigeria | Low | 4 | 32 | 78 | 4 to 11 | 0 | 0 | 0 | 0 |  | 0 | Yes | 0 |
| Zaman et al., 2008 | Pakistan | Low | 36 | n.r. | 375 | 5 half days | 0 | 0 | 0 | 0 |  | Yes | Yes | Yes |
| Zhang et al., 2007 | China | Med | 419 | n.r. | 696 | n.r. | Yes | 0 | Yes | Yes | Control of acute respiratory infections; Diarrheal disease control; Expanded plan of immunization & some nutrition intervention programmes, etc. | 0 | Yes | 0 |
