# Supplementary material for: Does Integrated Management of Childhood Illness (IMCI) Training Improve the Skills of Health Workers? A Systematic Review and Meta-Analysis
Source: PLoS One. 2013 Jun 12;8(6):e66030. doi: 10.1371/journal.pone.0066030 (PMC3680429; doi:10.1371/journal.pone.0066030)
Supplement: Text S1 — Protocol (version 4.3). Last updated on November 28th, 2012. (DOC) [file pone.0066030.s004.doc]

**Text S1. Protocol Version 4.3**

*Last updated on November 28^th^, 2012*

**Does the Integrated Management of Childhood Illness (IMCI) training improve the skills of health workers? A systematic review and meta-analysis**

**Background:**

Over 6.9 million children die annually in low and middle-income countries because of preventable and treatable diseases including diarrhea, pneumonia, malaria, and underlying malnutrition (UNICEF, 2011). In the mid 1990s, in an effort to reduce unacceptably high mortality and morbidity rates of children aged two months to five years, the World Health Organization, the United Nations Children’s Fund and their technical partners collaboratively developed the Integrated Management of Childhood Illness (IMCI). A strategy aimed at improving the quality of child health care (Arifeen et al., 2009, WHO 2003a), IMCI includes three components: strengthening the skills of health workers, improving the health system, and strengthening family and community practices (Duke, 2009). Over 100 countries have introduced or adopted components of the IMCI, although the level of intervention coverage varies markedly both within and between countries (Bryce et al., 2003; Duke, 2009).

The majority of focus has been on improving the skills of primary health workers (Lambrechts et al., 2004). Addressing health worker training is critical as many local practitioners may have limited formal medical training yet are often the first point of contact with health services (Arifeen et al., 2009). Furthermore, overlapping symptoms of many diseases (e.g., fever attributable to pneumonia vs. malaria) can make diagnosing and providing appropriate treatment difficult. For instance, studies have reported that in certain countries, less than 60% of health workers correctly prescribed oral antimicrobials (Armstrong et al., 2004; Amaral et al., 2004), and less than 10% of pneumonia cases were correctly diagnosed (Costello, 1997).

To improve the quality of care and case management practices, a comprehensive training program was developed for countries implementing IMCI. This includes evidence-based clinical algorithms, a pictorial counseling guide, and a one-page patient record for identifying and treating the leading causes of child death (Rowe et al., 2009). Furthermore, to enable standardized assessment and international comparative research, the WHO and UNICEF also developed the Health Facility Survey (WHO, 2003a) for evaluating IMCI.

A brief scouting review conducted in MEDLINE using the terms “Integrated Management of Childhood Illness*” or “IMCI” revealed approximately 200 published articles on the topic. In addition to a number of commentaries, position statements, and secondary analyses, approximately 40 peer-reviewed primary studies were available (Mason et al., 2009). These primary studies typically examined a subset of the IMCI training skills, and consisted of cluster randomized trials, pre-post studies, and cross-sectional studies, which were conducted in Latin/South America, the Middle East, Africa, and Asia. As a result, there may be heterogeneity regarding the effectiveness of IMCI training on health worker performance across these different regions, as there are different endemic levels of diseases, health practices, and economic resources available (Alberti, 1999).

To our knowledge, one narrative review and two systematic reviews are available on worker training. A narrative analytic review by the WHO (2003b) based on the results of five countries, broadly concluded that IMCI training improved health worker skills. Amaral & Victora (2008) further conducted a systematic review of the effect of training on healthcare performance up to 2006. However, of the 35 papers reviewed, 12 studies examined the sensitivity and specificity of IMCI algorithms for diagnosing diseases rather than on health worker performance per se. Since then, a number of evaluations have been completed, and recent studies suggest that not all facets of care improved equally with training (Arifeen et al., 2009). Most recently, Rowe and colleagues (2012) conducted a systematic review which examined whether shortening the training period from the IMCI’s standard 11-day protocol influenced performance outcomes. Therefore, we performed an updated systematic review and meta-analysis to examine whether IMCI training leads to improvements in the skills of health workers’ performance.

**Research Question:**

Primary Question: Do IMCI trained health workers have better performance than non-IMCI trained health workers in classifying illnesses, prescribing appropriate medications, vaccinating children with incomplete immunization records, and counseling caretakers on nutrition and administering oral therapies?

**Search Strategy:**

For our search, we intend to use different sources including peer-reviewed articles from online databases from PubMed, MEDLINE, Ovid Healthstar, CINAHL, Global Health, and EMBASE. To reduce publication bias, we will search relevant conference proceedings and abstracts. It is anticipated that many important reports and unpublished studies will not be found in the previously-listed sources; therefore, we will further search the grey literature via the World Health Organization Library Database (WHOLIS), WHO IMCI and the Multi-Country Evaluation (MCE) websites. We will also search the United Nations Children’s Fund (UNICEF), USAID, Department for International Development (DFID), Scopus, Proquest dissertations and theses, and Theses Canada Portal. Experts and research teams identified during the review process will be contacted for information regarding their data and/or knowledge regarding ongoing or unpublished studies. Finally, both reviewing authors will search the bibliography of all identified relevant publications.

In consultation with a research librarian, two search strings will be created to comprehensively identify publications on IMCI. The first string uses the English, French and Spanish names and acronyms of the intervention as text words and adjacent phrases: (“integrated management of childhood illness* [tw]” OR “IMCI [tw]” OR “prise en charge intégrée des maladies de l'enfant* [tw]” OR “PCIME [tw]” OR “Atención integrada a las enfermedades prevalentes de la infancia [tw]” OR “AIEPI [tw]”). The second string seeks to identify broader child health interventions that may have adopted components of IMCI as a part of their programs: (“Delivery of Health Care, Integrated [MeSH]” AND (“child health service* [tw]” OR “Child Welfare [MeSH]” OR “child nutrition science* [tw]” OR “child nutrition disorder* [tw]” OR “child* [tw]”)). Terms will be truncated to capture alternative spelling, and both search queries will be linked with the Boolean operator “OR” to expand the search. Because studies of public health interventions are often observational in design, no methodological search filters will be applied.

Based on the recommendations of a research librarian (D.L.), we will not include search strings for our target population (e.g., health workers) and outcome (e.g., health worker performance). During the pre-testing of a preliminary search strategy, which combined the search strings for the population, intervention, and outcome (PICO), we found that it failed to capture seminal studies related to IMCI-training and health worker performance. Although counter-intuitive, this may have occurred for a number of reasons. First, during our scout search, we were unable to identify comprehensive MeSH headings for our population or outcome, and thus we had to generate keywords and synonyms (e.g., community health workers, performance evaluation). However, we suspect that these lists of keywords were not exhaustive. As a result, when we combined the population and outcome search strings with the above intervention search string, we failed to capture the key articles. In comparison, when we used the intervention search string only, we were able to retrieve all of the seminal studies that were overlooked by the combined search strategy. Therefore, to provide the most comprehensive review possible, this strategy will use only the search string pertaining to IMCI.

**Identification of Articles for Eligibility of Systematic Review and Meta-analysis:**

The authors will conduct an initial review of the title and abstracts of the literature gathered, and we will determine if a full-text review is warranted.

Articles were included in the systematic review and meta-analysis if they fulfilled the following criteria:

1. Examined the performance between IMCI-trained and non-IMCI trained health workers.
2. Examined heath worker performance in any of the following Health Facility Survey indicators:
3. Child is correctly classified.
4. Child needing an oral antibiotic and/or an antimalarial is prescribed the drug correctly.
5. Child needing vaccinations leaves facility with all needed vaccinations.
6. Caretaker of sick child is advised to give extra fluids and continue feeding.
7. Child prescribed oral antibiotic and/or antimalarial and/or oral rehydration therapy whose caretaker is advised on how to administer the treatment.

Exclusion criteria included:

1. Articles that did not include patients aged two months up to five years.
2. Qualitative studies, case reports, case series, editorials, and commentaries.
3. Articles that only reported on the sensitivity and specificity of diagnostic algorithms, hospital-based IMCI clinical guidelines, and factors that influence health worker adherence to IMCI protocols.

Articles were included in the systematic review and not the meta-analysis if they:

1. Provided solely baseline data prior to IMCI implementation.
2. Used the same data set from a study that was already included in the systematic review.

The initial screen will be broad so that we may comprehensively capture as many relevant documents as possible. Agreement between reviewers will be recorded during the title and abstract review and during the full-text review. Any disagreement between reviewers will be resolved through discussion. In order to quantify the level of agreement between reviewers, Kappa statistics will be calculated following each review stage. For articles written in Chinese, French, or Persian, fluent research assistants will translate the documents for the two authors. Articles written in other languages will be translated via Google Translator. Again, both authors will review full-text documents, and any disagreement between reviewers will be resolved through discussion.

**Data Extraction**

A data extraction form was developed based on the Cochrane Effective Practice & Organization of Care Group (EPOC) Data Collection Templates for evaluating behavioural and educational interventions (Cochrane, 2002a). The proposed data extraction form contains seven sections (Appendix A). In addition we will document the study designs eligible for inclusion, characteristics of the study participants (e.g., health workers, child patients, and their caretakers), and whether the study was a part of the WHO Multi-Country Evaluation of IMCI. Among longitudinal studies, the most distal performance evaluation will be included in the primary analysis, and data from any preceding evaluations were retained for secondary analysis. We will also document the intra-class correlation coefficients to account for clustering among facilities and children assessed by a common health worker. Where possible, the most adjusted relative risks will be extracted, although unadjusted relative risks may be hand-calculated using the reported data. In instances where insufficient data were available for determining the relative risk or an equivalent point estimate, the corresponding authors will be contacted at least twice for supplemental data. If authors are unable to provide the necessary data, the study will be excluded from our meta-analysis.

For studies that do not report standard errors or confidence intervals, the standard error will be estimated using the following formula (Harris et al., 2008):


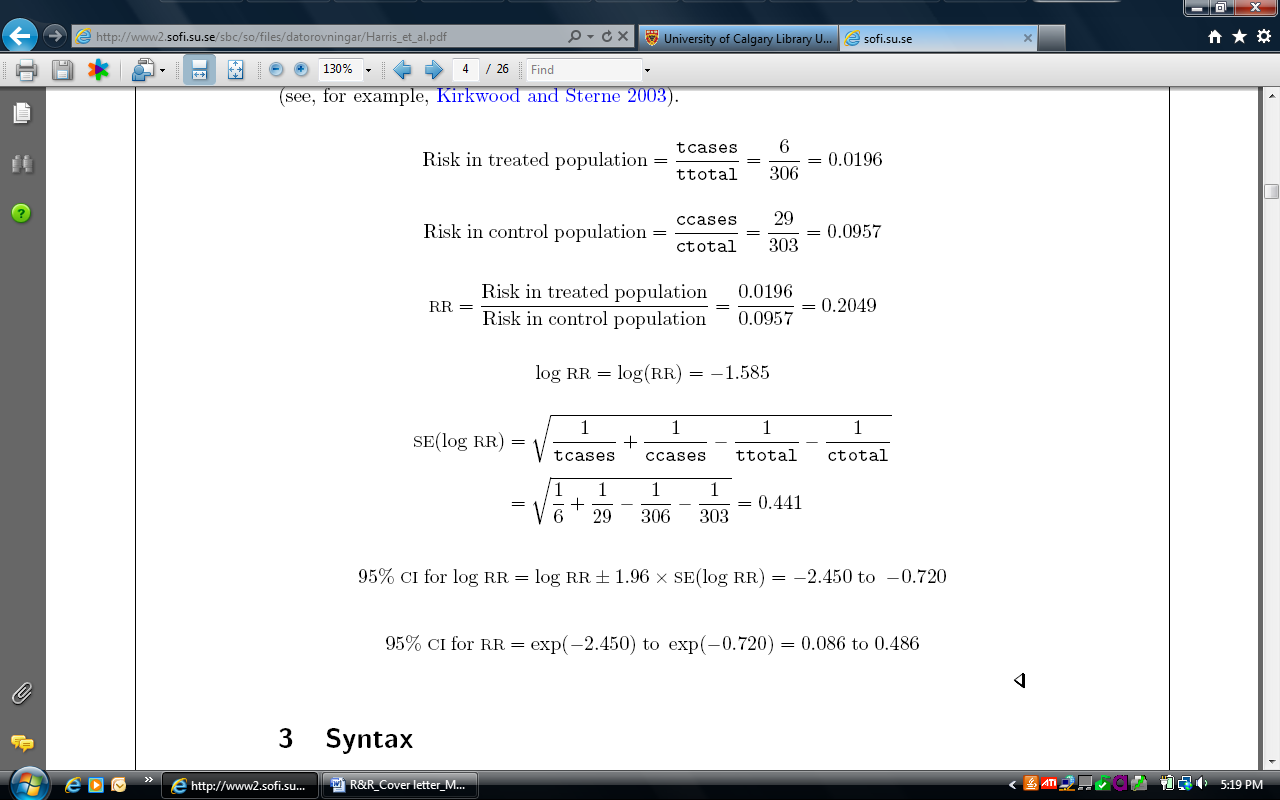


where *tcases* = cases in the treatment group, *ccases* = cases in the control group, *ttotal* = number in the treatment group, and *ctotal* = number in the control group.

Potential confounding variables and contributors to heterogeneity will be extracted including the training length, duration between training and performance evaluation, presence of concurrent interventions for child survival, whether health workers received at least one supervisory visit with observed case management in the previous six months, and whether sites had sufficient equipment (≥ 50% of recommended supplies) for delivering IMCI and vaccination programs (Appendix B). In addition, we will document as far as possible the presence of additional support and funding to strengthen IMCI; where available, we will extract multiple strata from within studies that compared health worker performance under standard IMCI with those receiving additional IMCI supports. We will further assess the influence of baseline health worker performance by dichotomizing studies using the median performance of workers not exposed to IMCI training. To account for broader social determinants of health, each study will be linked by country to their corresponding Human Development Index score (HDI), which is a well-established composite measure determined based on educational attainment, material wellbeing, and life expectancy.

We have also adopted the study quality criteria outlined in the Cochrane Handbook and the EPOC Data Collection Checklist (Cochrane, 2002b). Because randomized control trials, before and after studies, interrupted time series studies, and cross-sectional studies are eligible for inclusion, the extraction form has separate criteria for each. We will pay particular attention to the blinding of outcome assessors, comparability of groups at baseline, and adjustment for confounding. Any study quality criteria items that have been coded as unclear during the independent data extraction phase will be resolved through discussion by the two authors. In addition, we have identified a number of outcomes derived from the WHO’s Health Facility Survey (2003a, Appendix B); this standard assessment tool is used by the IMCI programs to assess the core indicators of quality care. Moreover, the units of measurement for each indicator are also standardized by the WHO, and are expressed as percentages as well as proportions (e.g., number of children treated correctly for a specific condition compared to the total number of children with that condition).

**Data Synthesis and Analysis**

Meta-analysis will be conducted using Stata version 12, and the “metan” command will be used to derive both the pooled relative risks and pooled risk differences. Given the variability in how the interventions were delivered and the diverse health and social contexts across studies, Dersimonian-Laird random-effect models based on the inverse-variance method will be used to summarize the effect estimates. To further contextualize our findings and assist in interpretation, we will calculate the number needed to treat for outcomes using the inverse of the pooled risk difference.

Because heterogeneity is anticipated in any public health meta-analysis, we will address this issue in accordance to current recommendations of exploring the underlying variables that drive heterogeneity rather than aborting the analysis (Lau et al., 1998). Heterogeneity will be explored through visual inspection of the forest plots and quantified using the I^2^ and Cochran’s Q statistic (significance of *p* < 0.05). Galbraith’s plots will then be used to qualitatively evaluate the contributions of individual studies to the heterogeneity metrics. Due to the small number of available studies, we decided a priori that we will not use multivariate meta-regression which would likely be underpowered. Instead, we will perform stratified analyses and univariate meta-regression to estimate the amount of heterogeneity attributable to these confounding variables. We will assess publication bias using the Begg’s test (significance of *p* < 0.05) and visual inspection of the funnel plots.

The inclusion of grey literature remains a contentious issue. Some authors have expressed concerns regarding the inclusion of grey literature in meta-analyses because of their potentially lower study quality (Sacks et al., 1996). To examine whether including grey literature may bias the results (Martin et al., 2005), we propose conducting a sensitivity analysis to examine the influence of peer-reviewed publications in addition to adjustment for potential confounding, use of blinding, and the comparability of groups at baseline. Because some studies provided only the stratified performance measures according to the presenting illnesses (e.g., pneumonia, malaria), we will analyze these data using the proportion of illnesses rather than the proportion of sick children that were correctly classified and managed. To create a single pair-wise comparison for each performance outcome, we will combine the illness strata by summing the number of illnesses correctly managed compared with the total number of presenting illnesses encountered by IMCI and non-IMCI health workers, respectively. Recognizing that the subset of patients with multiple concurrent illnesses will contribute to an overestimate of the relative risks, we will then perform a sensitivity analysis excluding those studies.

**References**

Alberti, P.W. (1999). Pediatric ear, nose, and throat services’ demands and resources: A global perspective. *International Journal of Pediatric Otorhinolaryngology, 49*(S1), S1-S9.

Amaral, J., Gouws, E., Bryce, J., Leite A.J.M., da Cunha, A.L.A., & Victora, C.G. (2004). Effect of Integrated Management of Childhood Illness (IMCI) on heath worker performance in Northeast-Brazil. *Cad Saude Publica, 20*(S2), S209-219.

Amaral, J.J.F. & Victora, C.G. (2008). The effect of training in Integrated Management of Childhood Illness (IMCI) on the performance and healthcare quality of pediatric healthcare workers: A systematic review. *Revista Brasileira de Saude Materno Infantil, 8,* 151-162.

Arifeen, S. E., Hoque, D., Akter, T., Rahman, M., Hoque, M. E., Begum, K., . . . Ahmed, S.

(2009). Effect of the Integrated Management of Childhood Illness strategy on childhood mortality and nutrition in a rural area in Bangladesh: A cluster randomised trial. *The Lancet, 374*(9687), 393-403.

Armstrong Schellenberg, J.R., Adam, T., Mschinda, H., et al. (2004). Effectiveness and cost of facility-based Integrated Management of Childhood Illness (IMCI) in Tanzania. *The Lancet, 364,* 1583-1594.

Bryce, J., Arifeen, S., Pariyo, G., Lanata, C.F., Gwatkin, D., Habicht, J. et al. (2003). Reducing child mortality: Can public health deliver? *The Lancet, 362*(9378), 159-164.

Costello, A. (1997). Integrated management of childhood illness. *The Lancet, 350,* 1266.

Cochrane Effective Practice & Organization of Care Group (EPOC) (2002a). Data Extraction Form. EPOC resources for reviewers. Available: [http://chmg.cochrane.org/sites/chmg.cochrane.org/files/uploads/Template-Data%20Extraction-CHMG.pdf](http://chmg.cochrane.org/sites/chmg.cochrane.org/files/uploads/Template-Data Extraction-CHMG.pdf). Accessed: September 29, 2009.

Cochrane Effective Practice & Organization of Care Group (EPOC) (2002b). Data Collection Checklist. EPOC resources for reviewers. Available: http://epoc.cochrane.org/sites/epoc.cochrane.org/files/uploads/datacollectionchecklist.pdf. Accessed: September 29, 2009.

Duke, T. (2009). Child survival and IMCI: in need of sustained global support. *The Lancet, 374,* 361-362.

Harris, R., Bradburn, M., Deeks, J., Harbord, R., Altman, D., & Sterne, J. (2008). Metan: Fixed-and random-effects meta-analysis. *Stata Journal, 8*(1), 3.

Lambrechts T, Bahl R, Robinson D, Aboubaker S, Picazo O. (2004). *The Analytic Review of the*

*Integrated Management of Childhood Illness Strategy*. WHO: Geneva. Available:

<http://www.who.int/maternal_child_adolescent/documents/9241591730/en/index.html>. Accessed October 10, 2012.

Lau, J., Ioannidis, J., Schmid, C. (1998). Summing up evidence: One answer is not always enough. The Lancet 351: 123-127.

Martin, J.L.R., Perez, V., Sacristan, M.. & Alverez, E. (2005). Is grey literature essential for a better control of publication bias in psychiatry? An example from three meta-analyses of schizophrenia. *European Psychiatry, 20,* 550-553.

Mason, E., Scherpbier, R., & Lawe-Davies, O. (2009). WHO position statement on IMCI. *The Lancet, 374*(9692), 782-783.

Rowe, A.K., Onikpo, F., Lama, M., Osterholt, D.M., Rowe, S., & Deming, M.S. (2009). A multifaceted intervention to improve health worker adherence to integrated management of childhood illness guidelines in Benin. *American Journal of Public Health, 99*(5), 837-846.

Rowe, A.K., Rowe, S.Y., Holloway, K.A., Ivanovska, V., Muhe, L., et al. (2012). Does

shortening the training on Integrated Management of Childhood Illness guidelines reduce its effectiveness? A systematic review. *Health Policy and Planning, 27*, 179-193.

Sacks, H.S., Reitmann, D., Pagano, D., & Kupelnick, B. (1996). Meta-analysis: An updated review. *Mount Sinai Journal of Medicine, 63,* 216-224.

UNICEF. (2011). Levels and trends of child mortality: Report 2011. Esitmates developmed by the UN inter-agency group for child mortality estimation. URL: <http://www.unicef.org/media/files/Child_Mortality_Report_2011_Final.pdf>. Accessed November 12, 2012.

World Health Organization. (2003a). Health facility survey: Tool to evaluate the quality of care delivered to sick children attending outpatient facilities. Available: <http://www.who.int/maternal_child_adolescent/documents/9241545860/en/index.html>

Accessed: October 1, 2009.

World Health Organization. (2003b). The analytic review of the integrated management of childhood illness strategy. Available: <http://www.who.int/child_adolescent_health/documents/9241591730/en/index.html>. Accessed: October 1, 2009.

**Appendix A: Data Extraction Form**

**1. Data Collection**

1.1 Review Information

| Name of Reviewer: | |
| --- | --- |
| Study Reference Number: | Date: |

1.2 Source Information

| Lead Author name: | | |
| --- | --- | --- |
| Title: | | |
| Journal: | | |
| Publication Year: | | Volume (Issue), Pages: |
| *If not peer-reviewed*, publication source: | | |
| Intervention Country: | Region/Continent: | |
| Language: | | |

**2. Study Eligibility Criteria**

2.1 Basic Inclusion Criteria

1. Reports on Primary Studies Yes  No  Unclear
2. Human subjects only Yes  No  Unclear
3. Children > 2 months – 5 years Yes  No  Unclear

2.2 Design Criteria

1. Randomized Control Trial Yes  No  Unclear
2. Cluster Randomized Control Trial Yes  No  Unclear
3. Controlled Before/After Design Yes  No  Unclear

d) Interrupted Time Series Yes  No  Unclear

e) Other design (specify): __________________

**3. Characteristics of the Intervention**

3.1 Type of Intervention

a) Standard 11-day IMCI Training Yes  No  Unclear

b) <11-day Modified IMCI Training Yes  No  Unclear  If yes, include duration of training (include units):

1. Estimated level of intended intervention coverage (include units & as much detail as possible):
2. Estimated level of achieved intervention penetration (include units & as much detail as possible):
3. Did the IMCI training include the usual supports (e.g., supervision training)?

Yes  No  Unclear  If yes, specify which supports were included?

1. Did the IMCI training include additional supports?

Yes  No  Unclear  If yes, what supports were included?

1. Type of health facility (describe & specify):

3.2 Type of Health Worker Performance Targeted by Intervention

*At least one of the following must be examined*

a) Assessing the Child Yes  No  Unclear

b) Vaccinations Yes  No  Unclear

c) Treating the Child Yes  No  Unclear

d) Counseling on fluids and/or feeding Yes  No  Unclear

e) Caregiver instructions Yes  No  Unclear

f) All of the above Yes  No  Unclear

**4. Characteristics of Participants**

4.1 Participating Health Workers

| **Characteristic** | **IMCI Health Workers** | **Control Health Workers** |
| --- | --- | --- |
| Sample Size of Health Workers |  |  |
| Age (SD) |  |  |
| Females # (%) |  |  |
| Nationality |  |  |
| Profession  Physicians # (%) |  |  |
| Nurses # (%) |  |  |
| Medical assistants # (%) |  |  |
| Nursing assistants # (%) |  |  |
| Welfare visitors # (%) |  |  |
| Others # (%) |  |  |
| Level of Health Worker Education  University # (%) |  |  |
| Post-secondary # (%) |  |  |
| Secondary # (%) |  |  |
| Primary # (%) |  |  |
| Less than Primary # (%) |  |  |
| Clinic/Practice Type (public/private) |  |  |
| Clinic/Practice Setting  Urban (%) |  |  |
| Rural (%) |  |  |

4.2 Participating Child Patients

| **Characteristic** | **IMCI Children** | **Control Children** |
| --- | --- | --- |
| Sample Size of Children |  |  |
| Age (SD) |  |  |
| Females # (%) |  |  |
| Nationality |  |  |
| Clinical Problem  Routine Check (%) |  |  |
| Presenting Illness (%) |  |  |
| Measles (%) |  |  |
| Diarrhoea (%) |  |  |
| Acute Respiratory Infection (%) |  |  |
| Malnutrition (%) |  |  |
| Malaria (%) |  |  |
| Measles & ARI (%) * |  |  |
| Measles & Diarrhoea (%)* |  |  |
| ARI & Diarrhoea (%)* |  |  |
| Other concurrent diagnosis (%) |  |  |

** Identified as occurring concurrently by Arifeen et al. (2009) in the Lancet.*

4.3 Participating Caretakers

| **Characteristic** | **IMCI Caretakers** | **Control Caretakers** |
| --- | --- | --- |
| Sample Size of Caretakers |  |  |
| Age (SD) |  |  |
| Females # (%) |  |  |
| Relationship to Child  Parent (%) |  |  |
| Other (%) |  |  |
| Nationality |  |  |
| Level of Caregiver Education  University # (%) |  |  |
| Post-secondary # (%) |  |  |
| Secondary # (%) |  |  |
| Primary # (%) |  |  |
| Less than Primary # (%) |  |  |

**5. Methods**

a) Unit of Allocation (if applicable):

b) Unit of Analysis (if applicable):

1. Estimated Power:
2. Specify data collection time points:

6. Outcomes Measures (derived from WHO)

| **Characteristic** ^¥^ | **IMCI** | **Control** |
| --- | --- | --- |
| Child is correctly classified danger signs |  |  |
| Proportion of sick children needed vaccinations (based on history) who leave the facility with all needed vaccinations (%, n/N) |  |  |
| Child needing an oral antibiotic and/or an antimalarial is prescribed the drug correctly (%, n/N) |  |  |
| Proportion of sick children whose caretakers are advised to give extra fluid/continue feeding (%, n/N) |  |  |
| Proportion of children prescribed ORS/antibiotic/antimalarial whose caregiver can describe correctly how to give the treatment (%, n/N) |  |  |

^¥^ *All outcome measures are derived from the IMCI Health Facility Survey (2003), a standardized evaluation procedure for IMCI programs (see definitions in Appendix B). The units of measurement are standardized by the WHO, and are expressed as percentages and as proportions of children treated correctly for a specific condition compared to the total number of children with that condition.*

7. OTHER INFORMATION

a) Was it an MCE study?

b) Data collection time points: Specify:__________

c) Health facilities with 60% IMCI trained HW (≥50%):

Yes  No  Unclear  If yes, specify:________

d) Supervisory observed visit in the last 6 months & case management (≥50%):

Yes  No  Unclear  If yes, specify:________

e) Facility has all equipment & supplies to support vaccination (≥50%):

Yes  No  Unclear  If yes, specify:________

f) Facility has all essential equipment and materials (≥50%)*

Yes  No  Unclear  If yes, specify:________

g) Complementary intervention implemented during same time as IMCI

Yes  No  Unclear  If yes, specify:________

h) IMCI training include additional supports**:**

Yes  No  Unclear  If yes, specify:________

i) Comparable baseline? Yes  No  Unclear  If yes, specify:________

j) Duration since training? Specify:________

k) Other (specify):

**7. EPOC Quality Criteria** (EPOC, 2002b)

7.1 Quality Criteria for Randomized Control Trials (RCTs and Cluster RCTs)

a) Concealment of allocation Yes  No  Unclear

b) Follow-up of health workers Yes  No  Unclear

c) Follow-up of patients Yes  No  Unclear

d) Blinded assessment of primary outcome(s) Yes  No  Unclear

e) Baseline measurement Yes  No  Unclear

f) Reliable primary outcome measure(s) Yes  No  Unclear

g) Protection against contamination Yes  No  Unclear

7.2 Quality Criteria for Controlled Before and After (CBA) Designs

a) Baseline measurement Yes  No  Unclear

b) Recorded characteristics of second control sites Yes  No  Unclear

c) Blinded assessment of primary outcome(s) Yes  No  Unclear

d) Protection against contamination Yes  No  Unclear

e) Reliable primary outcome measure(s) Yes  No  Unclear

f) Follow-up of professionals (exclusion bias) Yes  No  Unclear

g) Follow-up of patients Yes  No  Unclear

7.3 Quality Criteria for Interrupted Time Series (ITS) Designs

7.3.1 Protection against secular changes:

a) Intervention is independent of other changes Yes  No  Unclear

b) Data were analyzed appropriately Yes  No  Unclear

c) Reasons for number or pre/post measurement Yes  No  Unclear

d) Shape of intervention effect specified Yes  No  Unclear

7.3.2 Protection against detection bias:

a) Intervention unlikely to affect data collection Yes  No  Unclear

b) Blinded assessment of primary outcome(s) Yes  No  Unclear

c) Completeness of data set Yes  No  Unclear

d) Reliable primary outcome measure(s) Yes  No  Unclear

**Appendix B: Main outcome and additional variables definitions**

**Main Outcome Variables:**

**Classification = Child correctly classified (WHO, 2003a, pp. 156, 162)**

HFS Supplemental measure S5: Child is correctly classified.

Definition: Proportion of children whose classifications given by the health worker match all the classifications given by an IMCI-trained surveyor (validated classification).

How it is calculated:

Numerator = Number of children whose validated classifications (for the three major symptoms: cough, diarrhoea, and fever) matches the classifications given by the health worker

Denominator = Number of children seen

**Treatment/Medication = Child needing oral antimalarial and/or antibiotics and prescribed correctly (WHO, 2003a, p. 153)**

HFS Question 7: Child needing an oral antibiotic and/or an antimalarial is prescribed the drug correctly.

Definition: The proportion of children who do not need urgent referral, who need an oral antibiotic and/or an antimalarial who are prescribed the drug(s) correctly.

How it is calculated:

Numerator = Number of sick children with validated classifications, who do not need urgent referral, who need an oral anti- biotic and/or an antimalarial (pneumonia, and/or dysentery, and/or malaria, and/or acute ear infection, and/or anaemia in high malaria risk areas) who are correctly prescribed them, including dose, number of times per day, and number of days

Denominator = Number of sick children with validated classifications who do not need urgent referral, who need an oral antibiotic and/or an antimalarial

**Vaccination = Child needing vaccines leaves with all vaccinations (WHO, 2003a, p. 154)**

HFS Question 10: Child needing vaccinations leaves facility with all needed vaccinations.

Definition: The proportion of children needing vaccinations (based on vaccination card or history) who leave the HF with all needed vaccinations (according to national immunization schedule).

How it is calculated:

Numerator = Number of children who need vaccinations (based on vaccination card or history) who leave the HF with all needed vaccinations

Denominator = Number of children seen who need vaccinations (based on vaccination card or history)

**Nutrition = Caregiver was advised on fluids and/or feeding (WHO, 2003a, p. 154)**

HFS Question 9: Caretaker of sick child is advised to give extra fluids and continue feeding.

Definition: The proportion of sick children whose caretakers are advised to give extra fluid and continue feeding.

How it is calculated:

Numerator = Number of sick children with validated classifications, who do not need urgent referral, whose caretakers are advised to give extra fluid and continue feeding

Denominator = Number of sick children with validated classifications, who do not need urgent referral

**Instructing = Child was prescribed oral medications and caregiver was advised on how to administer (WHO, 2003a, p. 157)**

HFS Question S13: Child prescribed oral medication whose caretaker is advised on how to administer the treatment.

Definition: The proportion of children, who do not need urgent referral, who received or were prescribed an antibiotic and/or an antimalarial and/or ORS who received at least two treatment counseling messages.

How it is calculated:

Numerator = Number of children with validated classifications not needing referral, who do not need urgent referral, who received or were prescribed an antibiotic and/or an antimalarial and/or ORS who receive at least two treatment counseling messages (explanation on how to administer treatment, demonstration on how to administer treatment, open-ended question to check caretaker understanding)

Denominator = Number of children with validated classifications not needing urgent referral, who received or were prescribed an antibiotic and/or an antimalarial and/or ORS

**Additional Variables:**

**Health facilities with 60% IMCI trained HW (≥50%)* (WHO, 2003a, p. 155)**

HFS Question 18: Health facilities with at least 60% of workers managing children trained in IMCI.

Definition: The proportion of first level health facilities with at least 60% of health workers managing children trained in IMCI.

How it is calculated:

Numerator = Number of health facilities with at least 60% of health workers managing children who are trained in IMCI

Denominator = Number of health facilities surveyed

*We chose a cut-off of ≥50% of health facilities with 60% IMCI trained health workers

**Supervisory observed visit in the last 6 months & case management (≥50%)* (WHO, 2003a, p. 154)**

HFS Question 13: Health facility received at least one supervisory visit that included observation of case management during the previous six months.

Definition: The proportion of health facilities that received at least one visit of routine supervision that included the observation of case management during the previous six months.

How it is calculated:

Numerator = Number of health facilities visited that received at least one routine supervisory visit (supervisory visits do not include follow-up visits to health workers immediately after IMCI training) that included the observation of case management during the previous six months

Denominator = number of health facilities visited

*We chose a cut-off of ≥50% of health workers received at least one supervisory visit with observed case management in the last 6 months.

**Facility has all equipment & supplies to support vaccination (≥50%)* (WHO, 2003a, pp. 67, 155)**

HFS Priority indicator 16: Health facility has the equipment and supplies to support full vaccination services.

Definition: Proportion of health facilities that have the equipment and supplies to provide full vaccination services on the day of survey.

How it is calculated:

Numerator = Number of health facilities that have the equipment and supplies to support full vaccination services** on the day of the survey

Denominator = Number of health facilities surveyed

*We chose a cut-off of ≥50% of health facilities that has all equipment and supplies to support vaccination.

**Functioning refrigerator or cold chain, and functioning sterilizer (unless disposable needles and syringes are being used) and needles/syringes.

*Note:* In some programmes a ‘functional cold box’ is also considered an essential piece of cold chain equipment and is included in the definition. To be functional a cold box must be intact, have a fitting lid, and a complete rubber seal.

**Facility has all essential equipment and materials (≥50%)* (WHO, 2003a, pp. 158, 167)**

HFS Supplemental measure S17: Health facility has essential equipment and materials.

Definition: The proportion of health facilities that have all needed equipment and materials** available on the day of the survey.

How it is calculated:

Numerator = Number of health facilities with all needed equipment and materials (accessible and working weighing scales for adults and children, timing device, child health cards, source of clean water, spoons, cups and jugs to mix and administer ORS) available on the day of the survey

Denominator = Number of health facilities surveyed

*We chose a cut-off of ≥50% of health facilities that has all essential equipment and materials to support IMCI case management.

**Includes: accessible and working weighing scales for adult and children, timing device, child health cards, course of clean water, spoons and cups and jugs to mix and administer ORS, drug stock cards or logbook.

**Reported concurrent interventions/initiatives**

Definition: This includes non-IMCI interventions/initiatives that were implemented at the same time of IMCI implementation and that is aimed to improve the health of children under 5 years of age.

**IMCI training include additional supports**

Definition: This includes additional supports that are not typical to IMCI training, such as additional funding or automobiles for supervisors to travel to health facility sites.

**Is the research assessor blinded?**

Definition: This refers to whether the study stated if the data collector was blinded of the health worker’s previous training history (i.e., IMCI or non-IMCI trained).

**Comparable baseline**

Definition: This refers to whether the study presented comparable baseline characteristics regarding the health workers, patients, or location/context.

**Duration since training**

Definition: This refers to whether the study presented the time period between the completion of IMCI training and performance measured, with the cut-off time period of one year or greater for all IMCI trained health workers.

**MCE study**

Definition: This refers to whether the study was described as part of an IMCI Multi-Country Evaluation project.
